# Coxsackievirus infection induces direct pancreatic β cell killing but poor antiviral CD8^+^ T cell responses

**DOI:** 10.1126/sciadv.adl1122

**Published:** 2024-03-06

**Authors:** Federica Vecchio, Alexia Carré, Daniil Korenkov, Zhicheng Zhou, Paola Apaolaza, Soile Tuomela, Orlando Burgos-Morales, Isaac Snowhite, Javier Perez-Hernandez, Barbara Brandao, Georgia Afonso, Clémentine Halliez, John Kaddis, Sally C. Kent, Maki Nakayama, Sarah J. Richardson, Joelle Vinh, Yann Verdier, Jutta Laiho, Raphael Scharfmann, Michele Solimena, Zuzana Marinicova, Elise Bismuth, Nadine Lucidarme, Janine Sanchez, Carmen Bustamante, Patricia Gomez, Soren Buus, the nPOD-Virus Working Group, Sylvaine You, Alberto Pugliese, Heikki Hyoty, Teresa Rodriguez-Calvo, Malin Flodstrom-Tullberg, Roberto Mallone

**Affiliations:** ^1^ Université Paris Cité, Institut Cochin, CNRS, INSERM, Paris, France.; ^2^ Institute of Diabetes Research, Helmholtz Zentrum München, German Research Center for Environmental Health, Munich-Neuherberg, Germany.; ^3^ German Center for Diabetes Research (DZD e.V.), Neuherberg, Germany.; ^4^ Center for Infectious Medicine, Department of Medicine Huddinge, Karolinska Institutet, Karolinska University Hospital Huddinge, Stockholm, Sweden.; ^5^ Diabetes Research Institute, Leonard Miller School of Medicine, University of Miami, Miami, FL, USA.; ^6^ Department of Diabetes Immunology, Arthur Riggs Diabetes and Metabolism Research Institute, Beckman Research Institute, City of Hope, Duarte, CA, USA.; ^7^ Assistance Publique Hôpitaux de Paris, Service de Diabétologie et Immunologie Clinique, Cochin Hospital, Paris, France.; ^8^ Department of Diabetes and Cancer Discovery Science, Arthur Riggs Diabetes and Metabolism Research Institute, Beckman Research Institute, City of Hope, Duarte, CA, USA.; ^9^ Diabetes Center of Excellence, Department of Medicine, University of Massachusetts Medical Chan School, Worcester, MA, USA.; ^10^ Barbara Davis Center for Diabetes, University of Colorado School of Medicine, Aurora, CO, USA.; ^11^ Islet Biology Exeter (IBEx), Exeter Centre of Excellence for Diabetes Research (EXCEED), Department of Clinical and Biomedical Sciences, University of Exeter Medical School, Exeter, UK.; ^12^ ESPCI Paris, PSL University, Spectrométrie de Masse Biologique et Protéomique, CNRS UMR8249, Paris, France.; ^13^ Faculty of Medicine and Health Technology, Tampere University, Tampere, Finland.; ^14^ Paul Langerhans Institute Dresden (PLID), Helmholtz Munich, University Hospital and Faculty of Medicine, TU Dresden, Dresden, Germany.; ^15^ Assistance Publique Hôpitaux de Paris, Service d’Endocrinologie Pédiatrique, Robert Debré Hospital, Paris, France.; ^16^ Assistance Publique Hôpitaux de Paris, Service de Pédiatrie, Jean Verdier Hospital, Bondy, France.; ^17^ Department of Pediatrics, Division of Pediatric Endocrinology, Leonard Miller School of Medicine, University of Miami, Miami, FL, USA.; ^18^ Department of Immunology and Microbiology, Faculty of Health Sciences, University of Copenhagen, Copenhagen, Denmark.; ^19^ Indiana Biosciences Research Institute, Indianapolis, IN, USA.; ^20^ Fimlab Laboratories, Tampere, Finland.; ^21^ Department of Pediatrics, Tampere University Hospital, Tampere, Finland.

## Abstract

Coxsackievirus B (CVB) infection of pancreatic β cells is associated with β cell autoimmunity and type 1 diabetes. We investigated how CVB affects human β cells and anti-CVB T cell responses. β cells were efficiently infected by CVB in vitro, down-regulated human leukocyte antigen (HLA) class I, and presented few, selected HLA-bound viral peptides. Circulating CD8^+^ T cells from CVB–seropositive individuals recognized a fraction of these peptides; only another subfraction was targeted by effector/memory T cells that expressed exhaustion marker PD-1. T cells recognizing a CVB epitope cross-reacted with β cell antigen GAD. Infected β cells, which formed filopodia to propagate infection, were more efficiently killed by CVB than by CVB-reactive T cells. Our in vitro and ex vivo data highlight limited CD8^+^ T cell responses to CVB, supporting the rationale for CVB vaccination trials for type 1 diabetes prevention. CD8^+^ T cells recognizing structural and nonstructural CVB epitopes provide biomarkers to differentially follow response to infection and vaccination.

## Teaser

Coxsackievirus efficiently infects and destroys pancreatic β cells without eliciting strong CD8^+^ T cell responses.

## INTRODUCTION

Environmental factors weigh heavier than genetic predisposition in the pathogenesis of type 1 diabetes (T1D) (*1*) but remain elusive. Moreover, the increasing prevalence of neutral and protective human leukocyte antigen (HLA) class II haplotypes in the T1D population (*2*) and the parallel increase in disease incidence (*3*) suggest that environmental triggers are gaining importance. Such triggers are likely to exert their role early in life, as most children who subsequently develop T1D seroconvert for islet auto-antibodies (aAbs) before 2 years of age (*4*, *5*).

Infections by enteroviruses such as Coxsackieviruses B (CVBs) are suspected triggers of T1D (*1*). Key features relevant to this hypothesis are the virus capacity to infect pancreatic β cells, their high prevalence (>95% of the population is seropositive to at least one of the six CVB serotypes) and high incidence in infants and toddlers, and their oro-fecal transmission. CVBs are held as plausible candidates based on association studies in prospective cohorts of genetically at-risk children. These studies documented a temporal correlation between serological or molecular evidence of infection by some CVB serotypes and aAb seroconversion or clinical T1D onset (*6*). Using fecal metagenome sequencing, the Environmental Determinants of Diabetes in the Young (TEDDY) study (*7*) documented that the predisposing effect of CVB infections is on islet autoimmunity (i.e., aAb seroconversion) rather than on its subsequent progression to clinical T1D and that such predisposing effect is linked to prolonged rather than to short, independent CVB infections. These prolonged infections, identified by the protracted shedding of the same CVB serotype in sequential stool samples, are indicative of viral persistence (*8*), which may underlie a less effective antiviral immunity.

In parallel, spatial associations have been highlighted in histopathological studies on pancreas specimens (*9*–*11*). Positive staining for enteroviral VP1 protein was more prevalent in T1D than in nondiabetic donors and associated with the two histopathology hallmarks of T1D, namely, immune infiltration and HLA class I (HLA-I) hyperexpression. Only a minority of islets with residual β cells were VP1^+^, which may reflect low-grade infections persisting years after the initial CVB exposure.

Whether these associations underlie a cause-effect relationship remains unsettled. T1D primary prevention trials based on a multivalent inactivated CVB vaccine (*12*) are being considered (*11*). Such trials are needed to directly test whether preventing CVB infection affords protection from islet autoimmunity and subsequent T1D, which may support the pathogenic role of CVB. Several knowledge gaps, however, exist about the natural immune response against CVB (*1*). Available data are limited to serological studies, with one report suggesting that children who subsequently develop early anti-insulin aAbs lack anti-VP1 neutralizing antibodies (Abs) (*13*). In line with the TEDDY study (*7*), this could predispose to infections at higher viral loads, which may favor viremia and dissemination to the pancreas.

CD8^+^ T cells play a key role in the clearance of viral infections through cytotoxic lysis of infected cells, which process viral proteins and present peptides on surface HLA-I for T cell recognition (*14*). Against this background, it is unknown whether islet destruction and autoimmune initiation reflect a direct, primary β-cytolytic effect of viral infection or an indirect, secondary effect of antiviral T cells against infected β cells (*1*). The lack of knowledge about the CVB peptides processed and presented by infected β cells and recognized by CD8^+^ T cells hampers our possibility to understand this process and to follow response to infection and, eventually, vaccination. We aimed to fill these gaps by identifying the HLA-I–bound viral peptides presented by infected β cells and by using these peptides to track CVB-reactive CD8^+^ T cells and their cytotoxic activity against β cells.

## RESULTS

### CVB-infected β cells down-regulate surface HLA-I expression and present few selected HLA-I–bound viral peptides

**Fig. 1.**
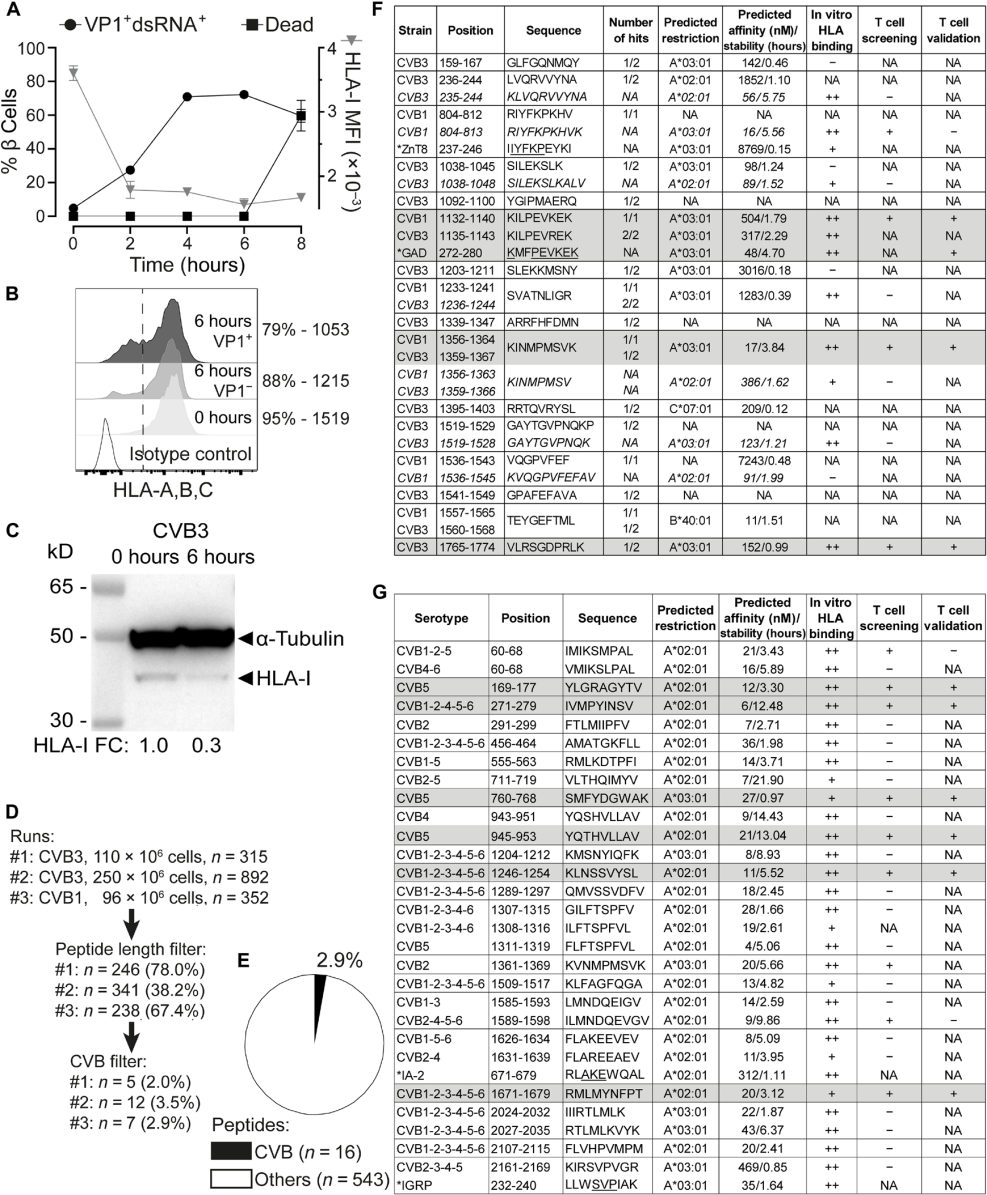
CVB-infected β cells down-regulate surface HLA-I and present few viral peptides. (**A**) Flow cytometry kinetics of viral VP1/dsRNA, cell death (left *y* axis), and HLA-I median fluorescence intensity (MFI; right *y* axis) in CVB3-infected ECN90 β cells (300 MOI). (**B** and **C**) Flow cytometry HLA-I expression on ECN90 β cells 6 hours postinfection (B; percent HLA-I^+^ cells and MFI indicated) and Western blot [C; HLA-I fold change (FC) normalized to loading control indicated]. (**D**) Three immunopeptidomics runs were performed at the 6-hour time point. Sequences were filtered for 8– to 12–amino acid length and for matches with the translated RNA of the CVB strains used; number and percent of peptides retained at each step are shown. (**E**) Percent CVB peptides out of total 8- to 12-amino acid peptides eluted. (**F**) CVB peptides retrieved from immunopeptidomics experiments (length variants of identified sequences in italics). The number of hits out of three peptidomics runs are listed for each peptide; see fig. S1 for mapping in the CVB polyprotein. Asterisks indicate CVB1_804–812_-homologous ZnT8_237–246_ and CVB1_1132–1140_/CVB3_1135–1143_-homologous GAD_272–280_ peptides (conserved amino acids underlined). NetMHCStabpan predicted affinity and stability values for the assigned HLA-I restrictions are listed. Subsequent columns list in vitro HLA binding results (see fig. S2) and peptides recognized by CD8^+^ T cells in the screening and validation runs (Fig. 2 and 3). T cell epitopes eventually validated are shaded in gray. CVB1_1132–1140_ and CVB3_1135–1143_ differ only by 1 amino acid and were counted as one peptide (total *n* = 16). NA, not applicable (or not assigned for predicted restrictions); +/++, positive; −, negative. (**G**) CVB nonamer peptides identified in a parallel in silico search for all six CVB strains. Asterisks indicate homologous β cell peptides retrieved by Blast. Three peptides not confirmed as binders in vitro were excluded (see fig. S2). For HLA-eluted peptides, sequence annotations refer to the CVB1/CVB3 strains used. For in silico predicted peptides, annotations indicate sequence identities across serotypes.

To identify the CVB peptides naturally processed and presented by β cells, we infected the human ECN90 β cell line. This line was derived from neonatal pancreas, expresses the β cell markers INS and PDX1 (*15*), the most prevalent HLA-I alleles HLA-A*02:01/A*03:01 (HLA-A2/A3 from hereon) along with T1D self-antigens (*16*), the Coxsackievirus/adenovirus receptor, and decay-accelerating factor required for CVB entry (*17*), and was previously used to identify autoimmune CD8^+^ T cell epitopes (*16*) and CD8^+^ T cell–mediated killing (*18*). In vitro infection with either CVB3 or CVB1 was highly efficient, leading to expression of VP1 and double-stranded RNA (dsRNA) in >70% of cells within 4 hours (Fig. 1A). Increased β cell death was apparent at 8 hours. For immunopeptidomics experiments, we selected the 6-hour time point, which corresponds to the infection plateau preserving viability. Notably, infected cells down-regulated HLA-I expression (Fig. 1, B and C).

Three preparations of ECN90 β cells (96 to 250 × 10^6^ cells each) infected with either CVB3 or CVB1 (Fig. 1D) were subsequently analyzed after purification of peptide (p)HLA-I complexes, peptide elution, liquid chromatography–tandem mass spectrometry (MS/MS), and bioinformatics assignment of MS/MS spectra (*16*). Together, a total of 16 unique CVB peptides were identified, which accounted for a minor fraction (2.9%; range, 2.3 to 3.8%) of the total 8 to 12 amino acid peptide display (Fig. 1, E and F). The β cell peptides identified in parallel did not yield any previously unidentified hit compared to our previous report (*16*). A search for putative transpeptidation products generated by the fusion of CVB and β cell peptide fragments using our previous scripts (*16*) did not return any unequivocal assignment.

Only 3 of 16 peptides (fig. S1) mapped to the larger structural capsid protein P1 (VP1-VP4); 5 of 16 mapped to the nonstructural protein P3 (3A-D). The smaller nonstructural P2 (2A-C) protein comprised most (8 of 16) of the eluted peptides. The percentage of eluted peptides derived from P2 were significantly more than expected (50% versus 26%), while few mapped to P1 (19% versus expected 35 to 39%; fig. S2, A and B). The eluted peptides were largely conserved across the 6 serotypes, and 5 of 16 (31%) were eluted from both CVB1- and CVB3-infected β cells.

This list of 16 peptides was complemented (i) by searching for length variants mapping to the same region of the eluted peptides (*16*) and displaying better HLA-A2/A3–binding score (*n* = 6; indicated in italics in Fig. 1F); (ii) by an in silico prediction of HLA-A2/A3–restricted peptides from representative strains of all six CVB serotypes (*n* = 28; Fig. 1G). Of note, CVB1_1356–1364_/CVB3_1359–1367_ (not listed in Fig. 1G) was the only HLA-I–eluted peptide that was also predicted in silico, supporting the complementarity of the two strategies.

Minor sequence homologies with β cell proteins (i.e., IA-2, IGRP, and ZnT8) were noted for some CVB peptides (Fig. 1, F and G), barring the eluted CVB1_1132–1140_ (KILPEVKEK) and CVB3_1135–1143_ (KILPEVREK), which displayed a high homology with a GAD_272–280_ peptide (KMFPEVKEK; conserved amino acids underlined) eluted from previous β cell preparations (*16*). Besides displaying a higher homology for GAD_272–280_, CVB1_1132–1140_ proved to be a better epitope in preliminary T cell analyses and was therefore retained for further studies.

We subsequently focused on predicted HLA-A2/A3–restricted peptides and validated their binding in vitro (fig. S2, C to E), leading to the final selection of 36 candidates for T cell studies (Fig. 1, F and G, second last column): 9 from immunopeptidomics experiments and 27 from in silico predictions; 24 HLA-A2–restricted and 12 HLA-A3–restricted.

Collectively, these results show that CVB-infected β cells down-regulate surface HLA-I expression and present few selected HLA-I–bound viral peptides, which significantly overlap between CVB1- and CVB3-infected cells, are largely conserved across serotypes and mostly map to the viral P2 protein.

### CVB peptides display limited recognition by circulating CD8^+^ T cells of CVB-seropositive individuals

**Fig. 2.**
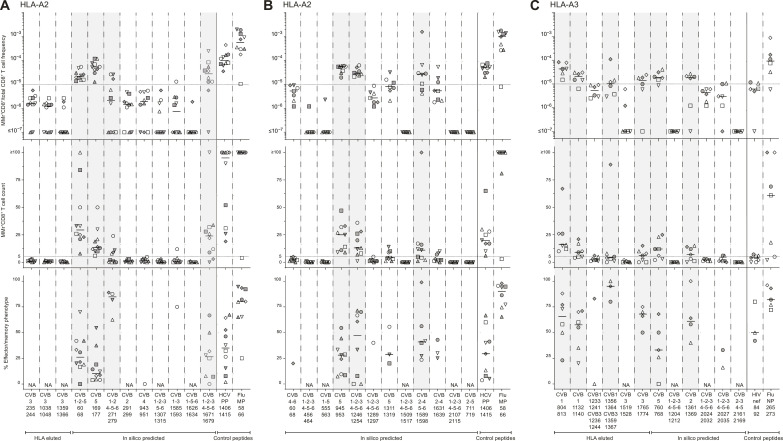
Screening of CVB peptides for recognition by blood CD8^+^ T cells in CVB-seropositive healthy adults. (**A** to **C**) HLA-A2–restricted (A and B) and HLA-A3–restricted candidates (C) were tested with combinatorial MMr assays [see reproducibility in fig. S2 (F and G)]. Each symbol represents a donor (legends in table S1), and the bars display median values. For each panel, the top graph depicts the frequency of MMr^+^CD8^+^ T cells out of total CD8^+^ T cells, with the horizontal line indicating the 10^−5^ frequency cutoff used as a first validation criterion; the middle graph displays the number of MMr^+^CD8^+^ T cells counted, with the horizontal line indicating the cutoff of five cells used to assign an effector/memory phenotype; the bottom graph shows the percent fraction of effector/memory cells (i.e., excluding naïve CD45RA^+^CCR7^+^ cells) among MMr^+^CD8^+^ T cells (for those donors with ≥5 cells counted; NA when not assigned). Peptides validated in this screening phase are highlighted in gray. In each panel, control peptides are derived from viruses eliciting predominantly naïve responses in these unexposed individuals (HLA-A2–restricted HCV PP_1406–1415_ and HLA-A3–restricted HIV nef_84–92_) and from influenza virus (Flu MP_58–66_ and NP_265–273_ peptides) eliciting predominantly effector/memory responses.

We next tested whether the candidate epitopes identified were recognized by circulating CD8^+^ T cells, using frozen/thawed peripheral blood mononuclear cells (PBMCs) from CVB-seropositive healthy adults (table S1). Combinatorial HLA-A2 or HLA-A3 multimer (MMr) assays (*16*, *18*–*20*) were used to probe up to 15 epitopes in the same blood sample, by staining each epitope-reactive T cell fraction with MMrs labeled with a unique pair of fluorochromes and subsequently deconvoluting each fraction by combinatorial gating (see details in fig. S3). The reproducibility was satisfactory for measuring both MMr^+^ T cell frequencies and naïve versus effector/memory phenotypes (inter-assay variability, 21 to 27%; fig. S2, F and G), and a detailed analysis of assay sensitivity is provided in fig. S4 and discussed further on. As these assays can probe up to 15 MMr specificities per sample, we screened candidate epitopes in three panels: two panels for HLA-A2–restricted candidates (*n* = 12 + 12; Fig. 2, A and B) and one panel for HLA-A3–restricted ones (*n* = 12; Fig. 2C). T cell frequencies segregated into two groups, above or below 1 MMr^+^/10^5^ total CD8^+^ T cells. This cutoff was therefore selected to retain candidate epitopes, as it corresponds to frequencies above the 1 to 10/10^6^ typically observed for most peptide-reactive naïve CD8^+^ T cells (*16*, *18*, *19*). Two additional peptides (HLA-A2–restricted CVB1-2-4-5-6_271–279_ and HLA-A3–restricted CVB1_1356–1364_/CVB3_1359–1367_) were retained because their cognate MMr^+^ cells, despite a frequency above this cutoff only in a donor subgroup, displayed a predominant (>60%) effector/memory phenotype (CD45RA^+^CCR7^−^, CD45RA^−^CCR7^+^, or CD45RA^−^CCR7^−^). Conversely, only some (6 of 11) peptides returning high cognate T cell frequencies displayed a similarly predominant effector/memory phenotype. Overall, 7 HLA-A2–restricted candidates (0 of 4 HLA-eluted and 7 of 20 in silico predicted) and 6 HLA-A3–restricted candidates (4 of 6 HLA-eluted and 2 of 6 in silico predicted) were retained. Given the high homology and cross-reactivity between the HLA-A3–restricted CVB1_1356–1364_/CVB3_1359–1367_ (KINMPMSVK) and CVB2_1361–1369_ (KVNMPMSVK), only the former (HLA-eluted) peptide was further considered.

**Fig. 3.**
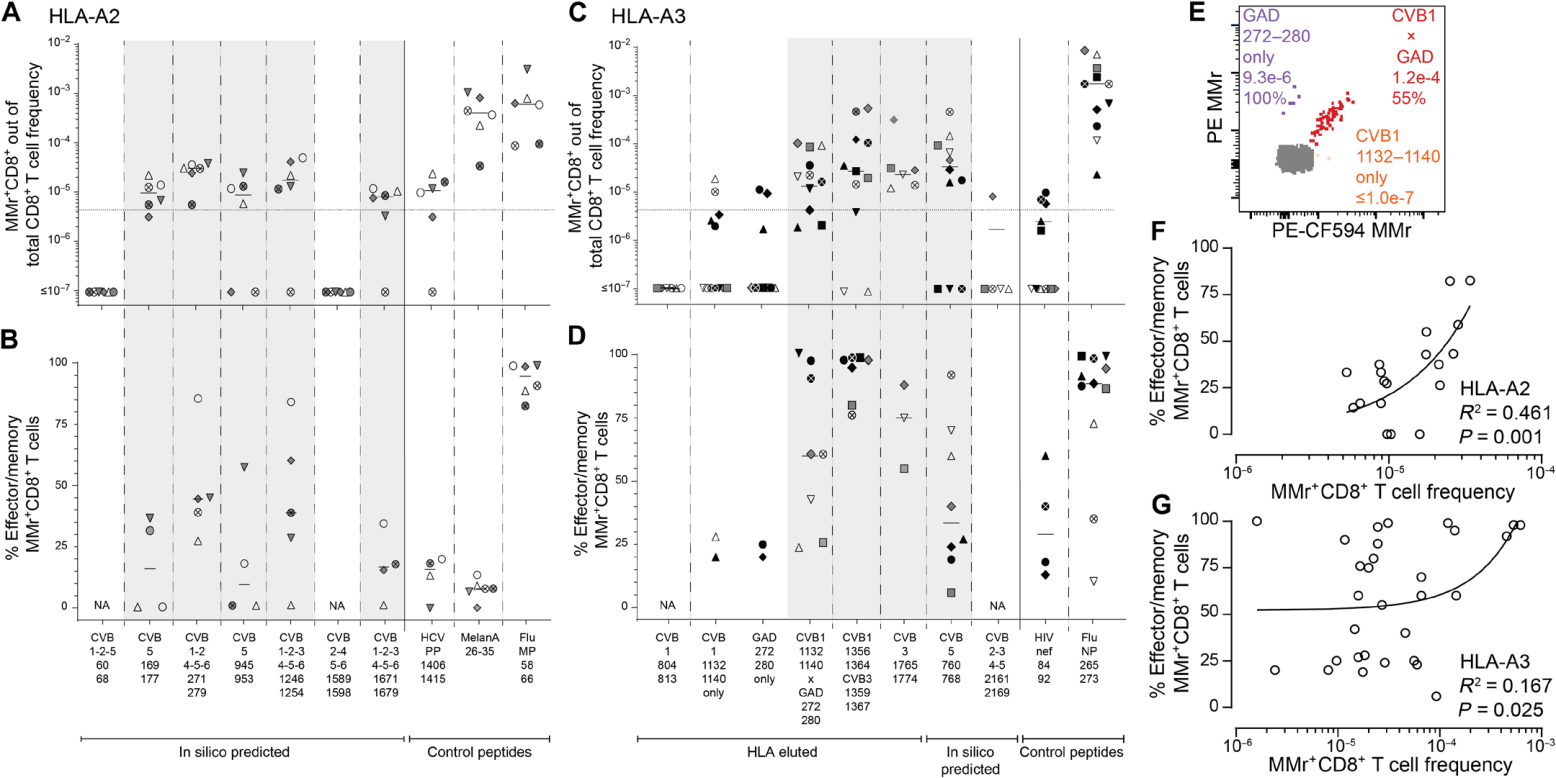
Validation of CVB epitopes as targets of blood CD8^+^ T cells in CVB-seropositive healthy adults. (**A** to **D**) The HLA-A2–rectricted and HLA-A3–restricted peptides selected for validation from the screening round (Fig. 2) are shown in (A) and (B) and (C) and (D), respectively (see fig. S3 for representative dot plots and table S2 for detailed results). In the HLA-A3 panel, the CVB2-3-4-5_2161–2169_ peptide not retained after screening was here included as negative control. Each symbol represents a donor (legends in table S1), with donors recruited in Paris and Miami depicted in (C) and (D) as white/gray and black symbols, respectively. Bars indicate median values. (A) and (C) depict the frequency of MMr^+^CD8^+^ T cells out of total CD8^+^ T cells, with the horizontal line indicating the 5 × 10^−6^ frequency cutoff used for validation, with only peptides scoring ≥3 MMr^+^ cells retained for this more stringent analysis. (B) and (D) show the percent fraction of effector/memory cells among MMr^+^CD8^+^ T cells. Validated peptides are highlighted in gray. In each panel, control peptides are derived from viruses or self-antigens eliciting predominantly naïve responses (HLA-A2–restricted HCV PP_1406–1415_/MelanA_26–35_ and HLA-A3–restricted HIV nef_84–92_) and from influenza virus, eliciting predominantly effector/memory responses. For the CVB1_1132–1140_ peptide homologous to GAD_272–280_, frequencies and effector/memory fractions are shown for T cells recognizing either peptide or both. (**E**) CD8^+^ T cells cross-reactive to CVB1_1132–1140_ (loaded on PE-CF594/BV711 MMrs) and GAD_272–280_ (loaded on PE/BV711 MMrs) appearing as a triple-stained population labeled by the four MMrs [see fig. S3 (C and D) for gating details]. (**F** and **G**) Correlation between the frequency of CVB epitope-reactive MMr^+^CD8^+^ T cells and their percent effector/memory fraction for HLA-A2– (F) and HLA-A3–restricted peptides (G). Each symbol represents an individual epitope specificity in an individual donor.

These 12 peptides underwent a second T cell validation round using more stringent gating and selection criteria. Representative dot plots obtained for these HLA-A2 and HLA-3 MMr panels are shown in fig. S3. Results are summarized in Fig. 3 and detailed in table S2. The frequency cutoff was set at 5 MMr^+^/10^6^ total CD8^+^ T cells to account for the more stringent gating criteria applied. Moreover, samples with low total CD8^+^ T cell counts were excluded to avoid any undersampling bias. Most peptides (5/7 for HLA-A2, 4/5 for HLA-A3; 9/12 in total) were lastly validated as relevant targets based on their high cognate T cell frequency. Of further note, HLA-A3^+^ donors were recruited at two different sites (Paris and Miami), and similar T cell frequencies were detected in the two subgroups (Fig. 3C).

The HLA-A3–restricted GAD_272–280_ peptide homolog to CVB1_1132–1140_ was further included at this stage to assess any potential cross-reactivity. To this end, we used our previous assay modification (*20*) in which the MMr pair loaded with CVB1_1132–1140_ (PE/CF594- and BV711-labeled) shared the BV711 fluorochrome with the MMr pair loaded with GAD_272–280_ (PE- and BV711-labeled), thus allowing to selectively gate CD8^+^ T cells recognizing only the CVB1 or GAD peptide or both. Most T cells displayed cross-recognition of the CVB1_1132–1140_/GAD_272–280_ homologous peptides, as shown by colabeling with the two MMr pairs (Fig. 3E and fig. S3, C and D) and by the higher frequencies of CVB1_1132–1140_/GAD_272–280_ MMr^+^ cells versus those recognizing either peptide alone (Fig. 3C).

Overall, 3 of 10 (30%) HLA-eluted peptides were validated as robust T cell targets. The recognition of a subset of naturally processed and presented peptides did not reflect an MS selection bias, as the same was observed with in silico predicted peptides (7/26, 27% validated as T cell targets). Moreover, as observed in T cell screening experiments, the effector/memory fraction was <50% in most individuals. This pattern contrasted with the higher frequency and near-complete effector/memory phenotype of control influenza virus–reactive CD8^+^ T cells, while it was similar to that observed for the naïve T cell epitopes hepatitis C virus (HCV) PP_1406–1415_ and HIV nef_84–92_ (all donors being HCV- and HIV-seronegative). Exceptions were noted for HLA-A3–restricted immunodominant CVB1_1356–1364_/CVB3_1359–1367_-reactive T cells and, to a lesser extent, CVB1_1132–1140_/GAD_272–280_–cross-reactive T cells (Fig. 3, C and D). Overall, CVB-reactive CD8^+^ T cell frequencies loosely correlated with the percent effector/memory fractions observed (Fig. 3, F and G), suggesting that higher frequencies may partly reflect prior in vivo expansion.

Collectively, these data show that a fraction of the few CVB peptides displayed by infected β cells is recognized by circulating CD8^+^ T cells of CVB-seropositive individuals and that only another subset is targeted by predominantly effector/memory T cells. Moreover, CVB1_1132–1140_-reactive T cells cross-recognize a homologous GAD_272–280_ sequence.

### CVB-reactive CD8^+^ T cells display similar features in the blood of adults and children

The modest CVB-reactive CD8^+^ T cell responses observed in CVB-seropositive healthy adults could reflect the long delay since CVB infections, which are more frequent during childhood. We therefore evaluated these responses in HLA-A2^+^ CVB-seropositive children, with or without T1D, recruited at two different sites (Miami and Paris; table S1). Given that PBMC numbers from pediatric donors are more limited and vary with age, we preliminarily defined the CV of T cell frequency measurements when decreasing PBMC numbers are sampled (fig. S4, A and B). The minimal total CD8^+^ T cell count maintaining a CV <35% was thus defined for each epitope and used to exclude pediatric samples with insufficient counts, thus limiting undersampling bias. As observed for CVB-seropositive healthy adults, children recruited at the two sites did not display major CD8^+^ T cell differences and were therefore analyzed altogether (fig. S4, C and D). Overall, the frequency of CVB-reactive CD8^+^ T cells detected in children fell in the same range of that of adult donors (Fig. 3) and was similar between T1D and healthy children, barring a marginally higher frequency of CVB5_945–953_-reactive T cells in the healthy group. The effector/memory fraction of CVB1-2-4-5-6_271–279_–reactive T cells was higher in T1D children. As in adults, influenza virus–reactive CD8^+^ T cells displayed higher frequencies and effector/memory fractions than CVB-reactive ones. A waning effect from remote CVB exposure is therefore unlikely to explain the limited magnitude of CVB-reactive T cell responses, as such responses were similar between healthy children and adults.

### CVB-reactive CD8^+^ T cells are also found in spleen and pancreatic lymph nodes and display a PD-1^+^ phenotype

**Fig. 4.**
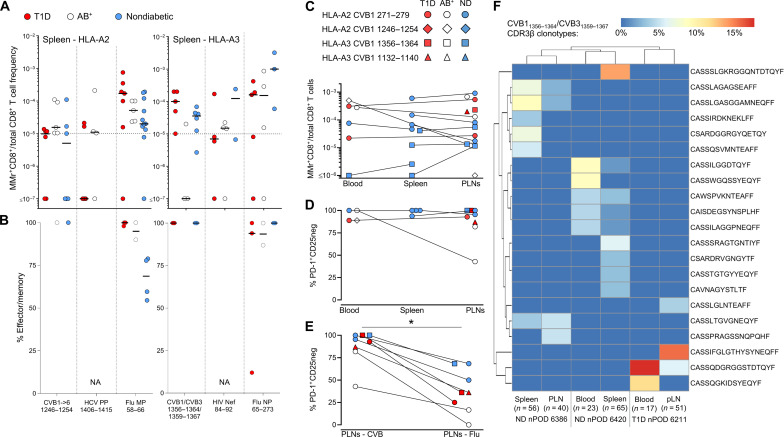
CVB-reactive CD8^+^ T cells are also found in spleen and PLNs and display a PD-1^+^ phenotype. (**A** and **B**) Detection of CVB epitope–reactive CD8^+^ T cells in splenocytes from nPOD organ donors (HLA-A2^+^, left; and HLA-A3^+^, right; see details in table S3). Each symbol represents a donor, and bars indicate median values. (A) depicts the frequency of MMr^+^CD8^+^ T cells out of total CD8^+^ T cells, with the horizontal line indicating the 10^−5^ frequency cutoff. A median of 73,320 CD8^+^ T cells were counted (range 9,611 to 307,697). Only epitopes testing positive are depicted (complete peptide panels listed in fig. S5A). (B) displays the percent effector/memory fraction within each MMr^+^CD8^+^ population for those donors/epitopes with ≥5 MMr^+^ cells counted. (**C** and **D**) CVB epitope–reactive CD8^+^ T cells in lymphoid tissues from nPOD cases. CVB MMr^+^CD8^+^ T cell frequencies (C) and percent PD-1^+^CD25^−^ MMr^+^ cells (D; for donors/epitopes with cell counts of ≥5) across available tissues are depicted. (**E**) Comparison of PD-1^+^CD25^−^ fractions between CVB and influenza virus MMr^+^CD8^+^ T cells detected in the same PLN; *P = 0.014 by Wilcoxon signed rank test. (**F**) Expanded TCR CDR3β clonotypes in individual CVB1/CVB3_1356–1364/1359–1367_-reactive CD8^+^ T cells from the same nPOD cases/tissues. Expanded clonotypes were defined as those found in ≥3 MMr^+^ cells in the same tissue from the same donor and are clustered according to frequency (one columns per tissue/case) and sequence similarity (one row per CDR3β clonotype). Further details are provided in figs. S5 and S6. ND, nondiabetic.

A second reason for the limited presence of circulating CVB-reactive CD8^+^ T cells could be sequestration in lymphoid organs. We therefore searched for these T cells in spleen cell samples from the Network for Pancreatic Organ Donors with Diabetes (nPOD; table S3 and fig. S5A). Given the small set of samples available, this analysis was not designed to test differences across T1D, aAb^+^, and nondiabetic donors but rather to assess whether the frequency and/or effector/memory phenotype was different compared to blood. Despite the limited T cell sampling imposed by the low cell numbers available, cognate T cells were detected in most donors for HLA-A2–restricted CVB1-2-3-4-5-6_1246-1254_ and HLA-A3–restricted CVB1_1356–1364_/CVB3_1359–1367_ (Fig. 4A). Compared to blood, the spleen harbored similar frequencies but higher effector/memory fractions (90 to 100%; Fig. 4B).

For HLA-A3^+^ T1D case #6480 displaying high CVB1_1356–1364_/CVB3_1359–1367_ reactivity (table S3), available pancreatic lymph node (PLN) cells expanded in vitro retrieved CD8^+^ T cells reactive to multiple CVB epitopes (fig. S5, B to E). We therefore searched for CVB-reactive T cells in the PLNs of nPOD organ donors and in the spleen and PBMC samples when available (table S3 and fig. S5A; representative staining in fig. S5F). CD8^+^ T cells reactive to a given CVB epitope displayed similar frequencies across tissues (Fig. 4C). CVB MMr^+^ frequencies were similar to those of influenza virus–reactive CD8^+^ T cells (fig. S5G). The vast majority of CVB-reactive T cells displayed an exhausted-like PD-1^+^CD25^−^ phenotype (Fig. 4D), which was instead more limited in control influenza virus–reactive CD8^+^ T cells (fig. S5H). Programmed death-1 (PD-1) was the most distinctive marker between CVB and influenza virus MMr^+^ cells across tissues (fig. S5I). When analyzed within the PLNs of the same donors, CVB-reactive CD8^+^ T cells were also significantly more PD-1^+^CD25^−^ than influenza virus–reactive ones (Fig. 4E). T cell receptor (TCR) sequencing of MMr^+^CD8^+^ T cells reactive to the most immunodominant CVB1_1356–1364_/CVB3_1359–1367_ sorted from paired samples highlighted the presence of expanded CDR3β clonotypes (Fig. 4F). This supports the prior in vivo expansion of these T cells, in agreement with the observed effector/memory phenotype. While some expanded clonotypes were shared across tissues from the same donor, we did not identify public clonotypes (i.e., shared across individuals). Expanded T cell clonotypes sequenced from PBMCs of CVB-seropositive donors were also exclusively private (fig. S6A). However, some common CDR3β and CDR3α motifs were noted (fig. S6B), along with preferential usage of TRBV6 (22%), TRBJ2-1 (22%), TRBJ2-7 (17%), TRBJ2-3 (15%), TRAV8 (19%), TRAV12 (15%) (fig. S6C), and the pairing of TRBV6 with TRAV8/TRAV12 (13% of total CVB1_1356–1364_/CVB3_1359–1367_ TCRs). Collectively, these results show that CVB-reactive CD8^+^ T cells are found in PLNs of T1D, aAb^+^, and nondiabetic donors and harbor expanded private clonotypes and an exhausted-like phenotype.

### CVB-reactive CD8^+^ T cells stained on spleen and PLN tissue sections are more abundant than other viral antigen reactivities in T1D donors

We examined more extensively the frequency and localization of CVB-reactive CD8^+^ T cells on frozen spleen, PLN, and pancreas sections from eight T1D, two double-aAb^+^, and five nondiabetic donors (table S4). In situ CD8 staining was combined with MMrs loaded with two CVB peptides, CMV/influenza virus peptides (one each; memory positive control), or West Nile virus (WNV) peptide (naïve negative control; fig. S7A). All MMrs were preliminarily validated by in situ staining on purified polyclonal CD8^+^ T cells (fig. S7B).

**Fig. 5.**
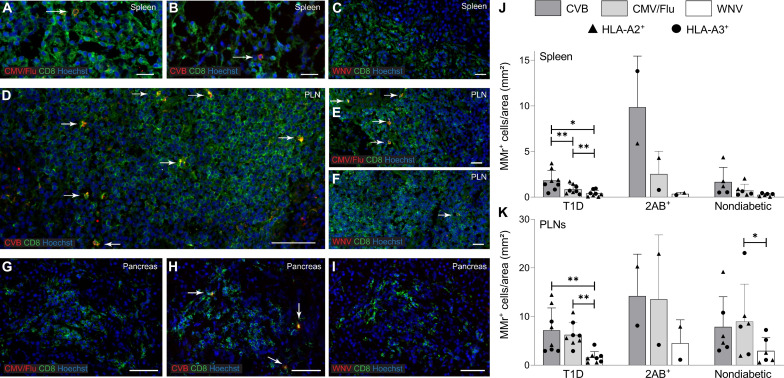
CVB-reactive CD8^+^ T cells stained on spleen and PLN tissue sections are more abundant than other viral antigen reactivities in T1D donors. (**A** to **I**) Representative immunofluorescence images of the spleen (A to C), PLN (D to F), and pancreas (G to I) tissue sections (detailed in table S4) stained with pooled CMV/Flu, pooled CVB or single WNV MMrs (red; see fig. S7), and CD8 (green). Cell nuclei are stained in blue. Scale bars: 20 μm for spleen, 50 μm for CVB PLN, 20 μm for CMV/Flu PLN, 10 μm for WNV PLN, and 50 μm for pancreas. White arrows indicate MMr^+^CD8^+^ T cells. (**J** to **K**) Bar graphs showing the mean + SD of MMr^+^ cell densities (number of MMr^+^ cells/mm^2^ area) in the spleen (J) and PLNs (K) for CVB, CMV/Flu and WNV specificities in T1D, double-aAb^+^ (2AB^+^), and nondiabetic HLA-A2^+^ (triangles) and HLA-A3^+^ (circles) donors. **P = 0.008 and *P = 0.05 by Wilcoxon signed rank test.

In the spleen (Fig. 5, A to C and J), CVB MMr^+^CD8^+^ T cells were more abundant than their CMV/influenza virus and WNV counterparts in T1D donors and in the 2 double-aAb^+^ donors available but not in the nondiabetic group. In PLNs (Fig. 5, D to F and K), densities were overall higher than in the matched spleens. CVB-reactive T cells were more abundant than WNV-reactive ones in T1D donors and in the two double-aAb^+^ donors available but not in the nondiabetic group. Control CMV/Flu MMr^+^CD8^+^ T cells were instead more abundant than for WNV in all groups. The overall concordance between CVB MMr^+^ frequencies measured by flow cytometry and CVB MMr^+^ densities quantified by tissue immunofluorescence was weak at low frequencies/densities but more consistent at higher values (i.e., in double-aAb^+^ donors) (fig. S7C).

Pancreas sections from the same donors displayed low numbers of islet-infiltrating CD8^+^ T cells in the T1D group, thus impeding an accurate quantification. However, very few or no islet-infiltrating cells were reactive to viral epitopes (Fig. 5, G to I). Accordingly, we did not find CVB-reactive CD8^+^ T cells in the pancreas when assessed by CVB peptide recall assays on carrier T cells transduced with TCRs sequenced from islet-infiltrating CD8^+^ T cells of T1D donors (*21*). Collectively, these results show that, while nearly absent in the pancreas, CVB-reactive CD8^+^ T cells are more abundant than other viral antigen reactivities in the spleen and PLNs of T1D donors compared to nondiabetic donors.

### CVB-infected β cells make filopodia and their subsequent death is primarily viral mediated

**Fig. 6.**
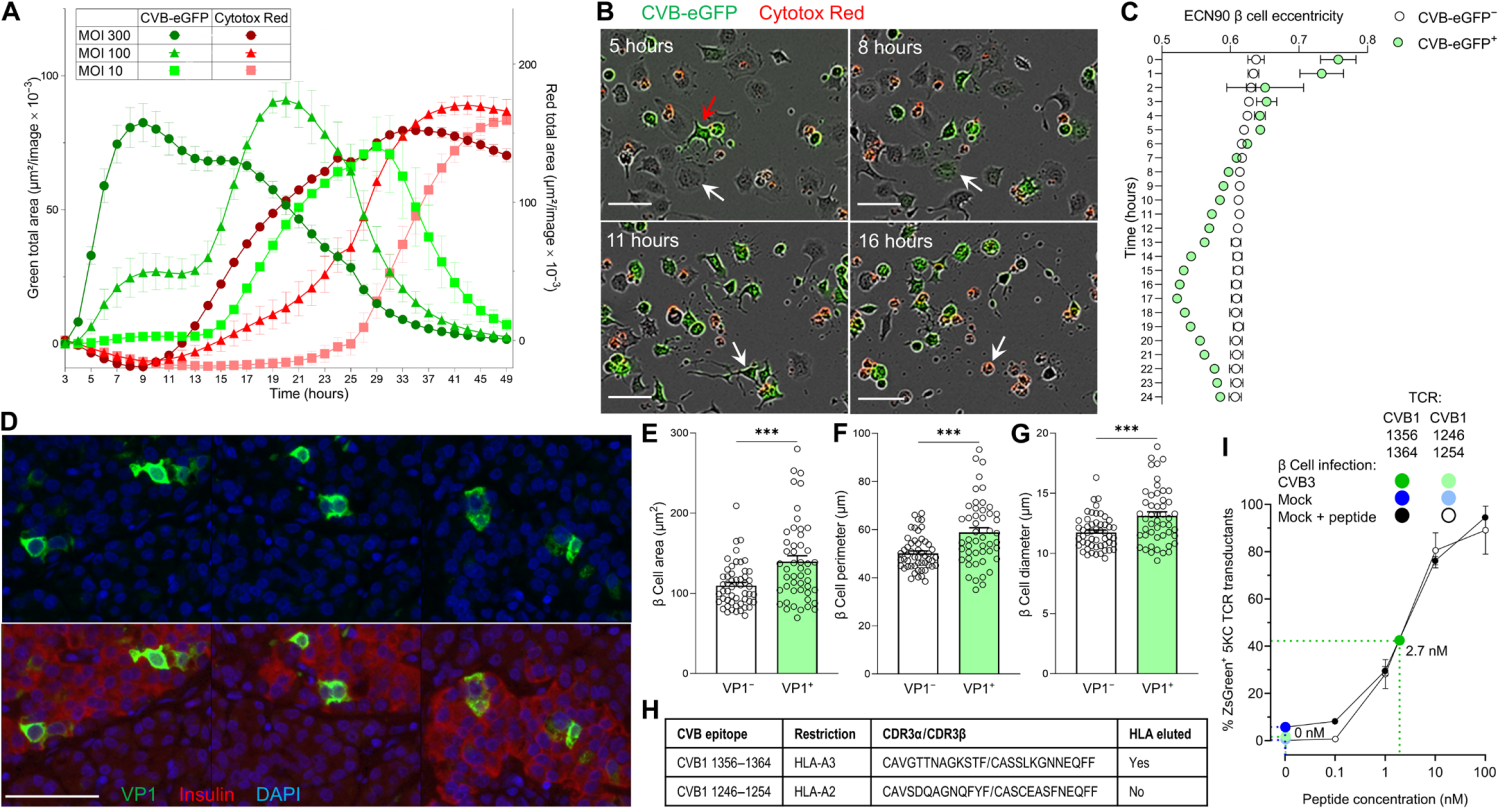
Kinetics of CVB infection and β cell death and presentation of CVB peptides. (**A**) Real-time imaging of infection and death (Cytotox Red) in CVB3-eGFP-infected ECN90 β cells. Infection and death curves are plotted as total area in green and red on the left and right *y* axes, respectively. Each data point represents mean ± SD of at least duplicate measurements (representative experiment performed in duplicate). (**B**) Representative images from movie S1 showing an intact β cell (5 hours, white arrow) touching the filopodia of an infected eGFP^+^ cell (red arrow), turning eGFP^+^ (8 hours) and subsequently emanating filopodia (11 hours) before dying (Cytotox Red^+^; 16 hours). Scale bars, 30 μm. (**C**) Eccentricity of infected versus noninfected ECN90 β cells (300 MOI). Each point represents mean ± SD of 8 images, *P* < 0.01 at all time points (barring 2 hours) by unpaired Student’s *t* test. (**D**) Representative pancreas sections from T1D donor 6243 stained for VP1 (green) and 4′,6-diamidino-2-phenylindole (DAPI; blue), alone (top) or with insulin (red, bottom). Scale bar, 50 μm. (**E** to **G**) Comparison of VP1^−^ versus VP1^+^ β cell area (E), perimeter (F), and diameter (G) in pancreas sections stained as above. Each point represents a β cell from sections of three T1D donors; bars represent mean + SEM. ***P ≤ 0.0004 by unpaired Student’s t test. (**H**) CVB-reactive TCRs reexpressed in carrier cells. (**I**) CVB1_1356–1364_-reactive TCR activation in NFAT-driven ZsGreen reporter, TCR-transduced 5KC cells upon an 18-hour coculture with CVB3-infected (dark green; 300 MOI) versus mock-infected ECN90 β cells (dark blue), plotted as mean ± 95% confidence interval of triplicate wells (representative experiment performed in duplicate). A parallel dose-response curve with increasing peptide concentrations pulsed on noninfected β cells (black) estimated the concentration of peptide recognized on CVB-infected β cells to 2.7 nM. A TCR recognizing the CVB1_1246–1254_ peptide (not eluted from β cells) is shown as negative control.

We next investigated the mechanisms of β cell death upon CVB infection. We first assessed the kinetics of CVB infection and β cell death in the absence of T cells using prolonged real-time imaging and a CVB3–enhanced green fluorescent protein (eGFP) strain (Fig. 6A). Both infection and death kinetics were accelerated with increasing multiplicities of infection (MOIs). At an intermediate 100 MOI, a first infection plateau was observed at ~12 hours, followed by a higher one at ~20 hours, possibly reflecting the spreading of new virions from a first pool of infected β cells to a new pool. At all MOIs, β cell death was complete ~20 hours after the infection peak. We also observed that intact β cells acquired CVB upon contact with filopodia protruding from infected cells, subsequently making filopodia themselves and infecting additional cells before dying (Fig. 6B and movie S1). Accordingly, the eccentricity (i.e., deviation from circularity to quantify cell elongation) of infected β cells varied over time, while it remained stable in noninfected cells (Fig. 6C). VP1^+^ β cells from pancreas tissue sections of T1D donors displayed a similar morphology (Fig. 6D) and higher cell area, perimeter, and diameter compared to VP1^−^ ones (Fig. 6, E to G).

To study how CVB-reactive CD8^+^ T cells contribute to β cell death, an immunodominant TCR clonotype recognizing HLA-A3–restricted CVB1_1356–1364_/CVB3_1359–1367_ (Fig. 6H) was reexpressed into carrier cells. TCR-transduced 5KC T cells exposed to CVB3-infected β cells up-regulated expression of a fluorescent nuclear factor of activated T cells (NFAT)-induced ZsGreen reporter (*22*), indicating display of the cognate peptide (Fig. 6I). A parallel dose-response standard curve using peptide-pulsed noninfected β cells estimated the concentration of CVB peptide naturally processed and presented by infected β cells to 2.7 nM. Thus, the natural CVB antigen presentation is sizable and sufficient to stimulate CVB-reactive T cells. A TCR recognizing HLA-A2–restricted CVB1-23-4-5-6_1246-1254_ (not eluted from β cells; Fig. 6H) did not elicit T cell activation upon contact with CVB-infected β cells in the absence of prior peptide pulsing (Fig. 6I).

**Fig. 7.**
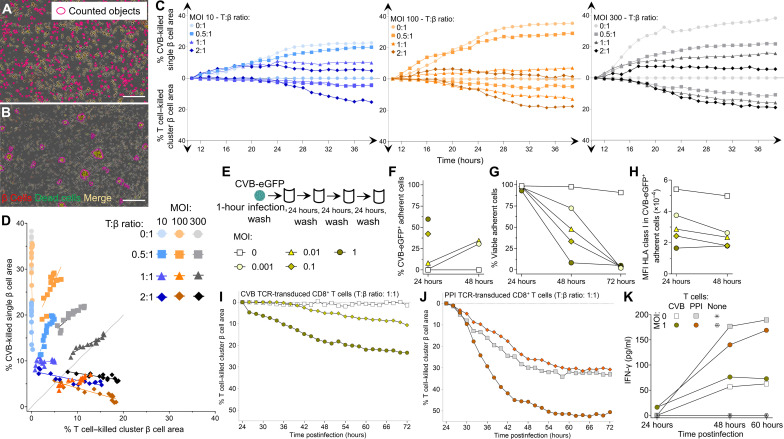
Infected β cells are more efficiently killed by CVB than by CVB-reactive T cells. (**A** and **B**) Representative real-time images from movies S4 and S5 (300 MOI, 1:1 T:β cell ratio) showing dead (Cytotox Green^+^) β cells (CellTracker Red^+^; dead β cells in yellow) killed by CVB (A) and CVB TCR-transduced CD8^+^ T cells (B), detected by single-cell and cluster analysis masks, respectively (details in movies S2 and S3). Scale bars, 30 μm. (**C**) Percent CVB-killed single β cell area (top) and T cell–killed cluster β cell area (bottom) at different MOIs and T:β cell ratios (ECN90 β cells stained as above), expressed as median percent total area from at least duplicate wells, normalized to the 8-hour time point (representative experiment performed in duplicate). (**D**) Correlation between percent CVB-killed and T cell–killed β cell area from (C) during the last 24 hours (details in fig. S8). (**E**) Low-grade infection protocol. ECN90 β cells were incubated with CVB3-eGFP for 1 hour, washed, and cultured for 72 hours, with washes to remove free virions, cell sampling for flow cytometry, and medium replenishment every 24 hours. (**F** to **H**) Percent CVB3-eGFP^+^ adherent ECN90 β cells (F), percent viable adherent cells (G), and HLA-I MFI (H). Each point represents the mean of duplicate measurements. For (F) and (H), some time points are not depicted due to the low number of remaining viable cells. (**I** and **J**) Percent T cell–killed cluster β cell area following 1-hour infection, washing, 24-hour culture, and real-time imaging after addition of CVB1_1356–1364_ (I) or PPI_15–24_ (J) TCR-transduced CD8^+^ T cells (1:1 T:β ratio). β cell areas are expressed as median percent of total β cell area from at least duplicate wells, normalized at the 24-hour time point [symbol legends as in (E), depicted in different colors for (J)]. (**K**) Mean IFN-γ secretion from the same duplicate wells.

Cytotoxic primary CD8^+^ T cells were then transduced with the same CVB1_1356–1364_/CVB3_1359–1367_ TCR and exposed to infected ECN90 β cells. Real-time imaging revealed that CVB-induced β cell death (i.e., in the absence of T cells) had distinctive morphological features compared to death induced by T cells (i.e., noninfected β cells pulsed with CVB peptide). While CVB-infected β cells died in a discrete, single-cell pattern (Fig. 7A and movie S2), those attacked by T cells formed clusters (Fig. 7B and movie S3). These distinct patterns allowed us to create single-cell and cluster cell analysis masks to differentially quantify CVB- and T cell–induced death, respectively (movies S4 and S5). Using this strategy, we compared the percentage of CVB-killed versus T cell–killed β cells over 48 hours, using increasing MOIs and T:β cell ratios (Fig. 7C). As expected, both types of cell death increased with time. While CVB killed β cells earlier, T cells started to do so only after ~24 hours. Starting from this time point, we therefore studied the relative contribution of CVB- and T cell–induced death (Fig. 7D and fig. S8A). CVB-mediated killing increased with higher MOIs not only in the absence of T cells but also in their presence for T:β cell ratios up to 1:1. T cell–mediated killing became predominant only at high T:β cell ratios of 2:1; however, this also reflected a decreased CVB-mediated killing. Since this decrease started already before 24 hours (Fig. 7C) and was equivalent when using nontransduced, non–CVB-reactive CD8^+^ T cells (fig. S8, B to D), it likely reflects reduced spreading of CVB from infected to intact β cells in the presence of high T cell numbers, independent of their cytotoxicity. There was no measurable infection of T cells. Collectively, these results show that most death of infected β cells is primarily induced by CVB. T cell–mediated cytotoxicity becomes predominant only at high numbers, which also reflects a steric limitation of CVB spreading by T cells, independently of their antigen reactivity.

To investigate whether low-grade CVB infection leads to similar outcomes, ECN90 β cells were infected with CVB-eGFP at low MOIs (0.001, 0.01, 0.1, and 1; Fig. 7E) and analyzed for the kinetics of infection, β cell death, and HLA-I modulation over 72 hours. Both infection (Fig. 7F) and death (Fig. 7G) increased with time in a MOI-dependent manner, with complete β cell death at all MOIs by 72 hours. HLA-I down-regulation was also observed at these low MOIs (Fig. 7H). We then infected β cells at 0.1 and 1 MOIs, and after 24 hours (when viability was still intact), we added CD8^+^ T cells transduced with either the previous CVB1_1356–1364_/CVB3_1359–1367_ TCR or a β cell–reactive HLA-A2–restricted preproinsulin (PPI)_15–24_ TCR (*23*) for real-time imaging. Also under this low-grade infection conditions, the killing mediated by CVB-reactive T cells was limited, plateauing at 23% (Fig. 7I), while PPI-reactive T cell–mediated killing was more extensive (Fig. 7J), thus excluding a general β cell resistance to cytotoxicity induced by CVB, e.g., by HLA-I down-regulation. Moreover, the higher MOI 1 enhanced cytotoxicity of both CVB- and PPI-reactive T cells but not their interferon-γ (IFN-γ) secretion (Fig. 7K), suggesting that this enhanced cytotoxicity reflects increased β cell vulnerability rather than increased T cell activation. IFN-α2a, IFN-β, and IFN-λ remained undetectable throughout the culture period.

Collectively, these in vitro results indicate that, upon infection, β cells are mostly killed directly by CVB, with a limited contribution of CVB-reactive CD8^+^ T cells. CVB infection can however enhance the β cell death mediated by autoimmune PPI-reactive CD8^+^ T cells.

## DISCUSSION

Despite lack of evidence for a causal relationship, CVB infections are gaining credit as candidate environmental triggers for T1D based on their temporal correlation with aAb seroconversion or clinical T1D (*6*, *7*) and on detection of pancreatic hallmarks of infection predominantly in patients with T1D (*9*–*11*). These associations have prompted a trial with the antiviral drugs pleconaril and ribavirin in new-onset T1D children and adolescents, which afforded some preservation of residual insulin secretion (*24*). Moreover, a multivalent CVB1-6 vaccine (*12*, *25*) led to a phase 1 trial in healthy adults with a PRV-101 vaccine covering the most prevalent CVB1-5 serotypes (NCT04690426). It is therefore relevant to address the knowledge gaps on CVB-induced immunity. While Ab responses have been extensively studied, T cell responses are poorly characterized (*1*). Such information would contribute to understanding the mechanisms of CVB-induced β cell damage and inform the development of vaccine trials. We focused on CD8^+^ T cells, which are key players in both antiviral responses and in the autoimmune attack against β cells. Two different, nonmutually exclusive scenarios could explain how CVB triggers T1D (*1*). First, weak anti-CVB immune responses could favor viral spreading and persistence, resulting in an enhanced, primary CVB-mediated killing of infected β cells. Second, an opposite scenario of strong antiviral immune responses could lead to indirect, secondary killing of infected β cells by viral-reactive T cells. In all cases, the final outcome would be the release of self-antigens in an inflammatory environment that may initiate/amplify autoimmunity, but the implications for vaccination strategies are different. While CVB vaccination may be beneficial to enhance weak immune responses and limit viral spreading, the second scenario may invite caution, as boosting antiviral T cell responses could trigger immune-mediated destruction of infected β cells.

The current study fills some of these gaps with several findings. First, an efficient immune escape mechanism mounted by CVB is down-regulation of surface HLA-I on infected β cells, as previously reported (*26*–*28*), which leads to the presentation of very few, selected CVB peptides. Most of them mapped to nonstructural protein P2, despite the fact that it lies downstream of structural P1 (VP1-VP4) in the viral polyprotein and is thus translated afterward. Nonstructural proteins may be better substrates for epitope display because they are not encapsulated and released with newly formed virions but eventually catabolyzed intracellularly, thus making them more readily accessible for antigen processing and presentation. Second, nonlytic CVB transfer through filopodia, previously described in other cells (*29*), may provide another immune escape mechanism by shielding virions from immune recognition and Ab-mediated neutralization. Nonlytic CVB spreading may further rely on other mechanisms, including the release of virion-loaded intracellular vesicles/autophagosomes (*30*) and the extrusion of live, infected cells from intact epithelial tissues (*31*).

Third, the overall strength of anti-CVB CD8^+^ T cell responses is limited in terms of number of epitopes recognized out of the few presented by β cells. While effector/memory responses were documented by surface phenotype and expanded TCR clonotypes, they were limited for most epitopes and donors, as supported by the low T cell frequencies observed, by a substantial naïve fraction, and by an exhausted-like PD-1^+^ phenotype, as observed in other persistent viral infections (*32*, *33*). This poor memory does not seem to reflect a long delay since CVB exposure, as T cell frequencies and effector/memory fractions in adults were similar to those in children, who are more frequently infected by CVBs. Moreover, poor memory generation seems universal, as similar CVB-reactive T cell frequencies and effector/memory fractions were observed in T1D and healthy children. This is not unexpected, in light of the late stage 3 disease of these patients with T1D. Studies on longitudinal samples from earlier T1D stages are needed to assess whether anti-CVB T cell memory is further diminished in children later developing islet aAbs, as observed in serological studies suggesting poor anti-CVB Ab responses (*13*, *34*). This could favor the prolonged pattern of CVB infections associated with islet aAb seroconversion (*7*). Footprints of CVB presence are also observed in T1D pancreata (*9*–*11*). On the other hand, the density of CVB-reactive CD8^+^ T cells was higher than that of other viral specificities in the spleen and PLN tissues of T1D donors, suggesting that, in the disease setting, these responses may be more efficiently elicited in secondary lymphoid organs, possibly in the context of higher CVB loads gaining access to the pancreas.

Collectively, these observations provide some rationale to induce immune memory through vaccination. Although CVB-reactive CD8^+^ T cells were capable of killing infected β cells, their role was predominant only at the highest T:β cell ratios and was favored by a concomitant steric inhibition of CVB transfer. Killing was otherwise mainly CVB mediated, even at low MOIs, suggesting that autoimmune triggering predominantly relies on the direct, primary cytotoxic effect of the virus releasing islet antigens. In line with these observations, CVB-reactive CD8^+^ T cells were near-absent in islet infiltrates. These results suggest that vaccination is unlikely to enhance β cell destruction, as ruled out in rhesus macaques (*12*). In addition, the multivalent PRV-101 CVB vaccine is produced with chemically inactivated mature virions that do not comprise the nonstructural peptides preferentially presented by β cells, including the CVB1_1132–1140_/CVB3_1135–1143_ epitope cross-reactive with the endogenous β cell peptide GAD_272–280_. This peptide maps to a larger GAD region, whose CVB cross-reactivity initially reported (*35*) was subsequently dismissed (*36*, *37*). We now show that CVB/GAD cross-recognition is at play for CD8^+^ T cells, with the large majority of them reactive to both epitopes. To better understand the pathogenic mechanisms of CVB infection, it will be important to test whether these cross-reactive T cells can lyse noninfected β cells. However, the opposite possibility that these CVB/GAD cross-reactive responses may actually provide better antiviral protection should not be dismissed (*38*). Anti-GAD aAbs are more prevalent at older ages (*39*) and have been suggested to be protective, as children with multiple aAbs, including anti-GAD, have a lower risk of clinical progression than those without anti-GAD (*38*).

By combining immunopeptidomics and in silico predictions, we identified several other epitopes that, although not presented by β cells, were recognized by CD8^+^ T cells. These two in vitro and in silico strategies proved complementary, yielding different sets of peptides and validated T cell epitopes that overlapped only for CVB1_1356–1364_. The T cell epitopes missed by immunopeptidomics may reflect in vivo T cell expansion elicited by other infected cells or antigen-presenting cells. Those missed by prediction algorithms exemplify the limitations of relying exclusively on HLA-I binding affinity/stability. Overall, we identified two epitopes for each of the HLA-A2 and HLA-A3 restrictions studied that displayed high immunodominance and immunoprevalence: HLA-A2–restricted CVB1_271–279_, CVB1_1246–1254_ and HLA-A3–restricted CVB1_1356–1364_ and, to a lesser extent, CVB1_1132–1140_. A confounding effect of polio vaccination is unlikely, in light of their limited homology with the poliovirus serotypes of the vaccine (fig. S9A), which only comprises structural proteins like the PRV-101 CVB vaccine. One reason for their preeminence may instead be their conservation across CVB serotypes. A larger panel of epitopes (fig. S9B) mapping to structural P1 (VP1-4) (e.g., HLA-A2–restricted CVB5_169–177_, CVB1_271–279_; HLA-A3–restricted CVB5_760–768_) and nonstructural P2-P3 (e.g., HLA-A2–restricted CVB1_1246–1264_, CVB1_1671–1679_; HLA-A3–restricted CVB1_1132–1140_, CVB1_1356–1364_, CVB3_1765–1774_) could be used to differentially monitor T cell responses to natural infection (where both structural and nonstructural epitopes should be targeted) versus those elicited by vaccination (with only structural epitopes recognized), analogous to what is routinely done for hepatitis B virus (by testing anti-HBsAg versus anti-HBcAg Abs) and severe acute respiratory syndrome coronavirus 2 (by testing antispike versus antinucleoprotein Abs). This distinction cannot be made by CVB serology.

The surface HLA-I down-regulation induced in CVB-infected β cells decreased the presentation not only of CVB peptides but also of endogenous ones. Only 152 to 206 8- to 12-amino acid human peptides were retrieved from each biological replicate (96 to 250 × 10^6^ cells) as compared to the 3544 peptides retrieved using equivalent or lower total cell numbers (100 × 10^6^) of untreated β cells in our previous work (*16*). However, besides causing β cell lysis and self-antigen release, CVB infection triggers a type I IFN response (*1*), which may create an immunogenic environment and induce the secondary HLA-I hyperexpression colocalized with viral VP1 protein in islets of patients with T1D (*10*) and persisting years after clinical diagnosis (*40*). This HLA-I hyperexpression leads to an increased peptide presentation (*16*), thus enhancing β cell vulnerability to autoimmune T cells. While we observed an enhanced cytotoxicity of PPI-reactive CD8^+^ T cells against CVB-infected β cells, this enhancement was not paralleled by an increased IFN-γ secretion nor by any detectable IFN-α2a, IFN-β, or IFN-λ, suggesting that other potentiation mechanisms may be at play. These in vitro results should however be interpreted with caution, as IFN responses may originate not only from T cells (IFN-γ) and β cells (mainly IFN-β) but also from plasmacytoid dendritic cells (mainly IFN-α) responding to DNA from dying β cells (*41*) and, possibly, viral RNA. Multivariate in vitro systems, e.g., using pancreas tissue slices, are needed to more accurately model these interactions. However, IFNs may be insufficient at mitigating CVB evasion from HLA-I presentation and rather favor their persistence hidden inside β cells. The group of Whitton (*42*) used in vivo mouse models to elegantly show that a recombinant CVB3 encoding highly immunogenic lymphocytic choriomeningitis virus (LCMV) epitopes barely activates LCMV TCR-transgenic T cells, suggesting near-complete evasion from MHC class I presentation even when IFN responses are operational. This is at variance with a recent work documenting a protective role of these CD8^+^ T cell responses against CVB-induced lethality (*43*). The dominance of a primary CVB-mediated cytopathic effect over secondary T cell–mediated lysis that we observed may be specific to human β cells and reflect their exquisite sensitivity to viral stress (*44*).

This study carries limitations. First, only the peptides naturally processed and presented by β cells were considered. While this allowed us to directly address the possibility that vaccination may trigger CD8^+^ T cell cytotoxicity against β cells, the peptide display offered for T cell activation by other cells, including antigen-presenting cells, may be broader, as suggested by the identification of additional epitopes by in silico predictions. Notably, it will be important to define the peptides displayed by infected enterocytes at the viral entry site, by dendritic cells and macrophages that may take up antigens locally and in draining lymph nodes, and upon ensuing IFN responses. Second, the contribution of CD4^+^ T cell responses to CVB immunity remains to be explored. Third, it is possible that the interplay between CVB, β cells, and T cells described herein may change if the primary infection evolves toward a chronic, slowly replicative persistence in β cells, e.g., by naturally occurring 5′-terminal deletions of the CVB genome (*45*).

In conclusion, the identification of the CVB epitopes targeted by CD8^+^ T cells allowed us to define the role of CVB infection in mediating primary β cell lysis while inducing limited antiviral CD8^+^ T cell responses, which mitigates the risk that CVB vaccination may precipitate the destruction of infected β cells. The poor CD8^+^ T cell memory against CVB lends rationale for inducing such memory through vaccination.

## MATERIALS AND METHODS

### Experimental design

The objective of this study was to identify the HLA-I–bound peptides presented by CVB infected β cells and the CD8^+^ T cells recognizing them. Hypotheses were formulated on a prospective basis guided by the data. On the basis of a detailed analysis of the CVB peptides identified and of their cognate CD8^+^ T cells, we initially hypothesized that CVB infection induces a limited peripheral CD8^+^ T cell immunity. We excluded that this was due to a waning effect from remote CVB exposure, as such responses were similar in children and adults. We confirmed the effector/memory phenotype of a fraction of these CD8^+^ T cells based on surface biomarkers and on the presence of expanded TCR clonotypes. In the blood, these CVB-reactive CD8^+^ T cells displayed a lower frequency than other viral epitope-reactive CD8^+^ T cells and no distinctive features between T1D and healthy donors. We hypothesized that the limited presence of circulating CVB-reactive CD8^+^ T cells could reflect sequestration in lymphoid organs. We confirmed this hypothesis by showing that CVB-reactive CD8^+^ T cells are more abundant than other viral antigen reactivities in the spleen and PLNs of T1D donors. On the basis of these results, we lastly hypothesized that the death of infected β cells is primarily induced by CVB itself rather than by antiviral T cell responses. We confirmed this hypothesis by real-time imaging. Samples were processed in batch, and no outliers were excluded. All in vitro experiments were performed on at least two separate occasions. For ex vivo MMr analyses, undersampled data points were excluded.

### Preparation and titration of CVB1 and CVB3 stocks

CVB1 [American Type Culture Collection (ATCC) VR-28 strain] and CVB3 (Nancy strain) were grown in HeLa cells. Supernatants and cells were collected after overnight culture, subjected to 3 freeze/thaw cycles, centrifuged at 4000 rpm, and further purified by ultracentrifugation. Virus stocks were titrated on HeLa cells and used for infection without further passaging. Next-generation sequencing was used to confirm serotypes and identify sequence variants (quasi-species). CVB3-eGFP (*46*), originally obtained as a plasmid, was generated by transfection of HeLa cells. The cell supernatant was collected and used to produce a new virus stock in HeLa cells. After overnight culture, cells and the supernatant were collected, freeze/thawed thrice, centrifuged, titrated, and used as above.

### In vitro infection

The ECN90 β cell line (HLA-A*02:01/03:01, HLA-B*40:01/49:01, and HLA-C*03:04/07:01) (*15*, *18*) was cultured in Dulbecco’s modified Eagle’s medium (DMEM)/F12 Advanced medium (Thermo Fisher Scientific) supplemented with 2% bovine serum albumin (BSA) fraction V, fatty acid free (Roche), 50 μM β-mercaptoethanol (Sigma-Aldrich), 10 mM nicotinamide (Merck), sodium selenite (1.7 ng/ml; Sigma-Aldrich), penicillin-streptomycin (Gibco), and 1% glutaMAX (Gibco). Cells were seeded at 16.5 × 10^6^ cells in 150-cm^2^ culture flasks (TPP) coated with 0.25% fibronectin from human plasma (Sigma-Aldrich) and 1% extracellular matrix from Engelbreth-Holm-Swarm murine sarcoma (Sigma-Aldrich) and cultured at 37°C in 5% CO_2_ for 16 to 24 hours. They were infected with 300 MOI of CVB1 or CVB3 for 1 hour in BSA-free supplemented medium. After gentle washing, the culture was pursued for 6 hours, followed by trypsinization, washing with phosphate-buffered saline (PBS) and processing for flow cytometry or freezing at −80°C as dry pellets for peptidomics experiments.

Infection was verified on cells stained with Live/Dead Violet (Thermo Fisher Scientific) and monoclonal Abs (mAbs) to HLA-A,B,C (RRID:AB_2917755), VP1 (clone 3A6) (*47*), dsRNA (RRID:AB_2651015) followed by a fluorescein isothiocyanate (FITC)–conjugated mouse anti-rat immunoglobulin G (IgG; RRID:AB_465229) and an Alexa Fluor (AF) 594–conjugated goat anti-mouse IgG (RRID: AB_2762825). Western blot for HLA-A,B,C was performed on whole-cell lysates using the mAb HC10 (RRID: AB_2728622; produced in-house) and a mAb to α-tubulin (RRID: AB_1210457) as loading control, followed by a horseradish peroxidase–conjugated goat anti-mouse IgG (RRID: AB_2728714) and Pierce ECL reagents (Thermo Fisher Scientific).

### Isolation and identification of HLA-I–bound peptides

Frozen cell pellets were lysed for 1 hour at 4°C with 1% (w/v) octyl-β-d-glucopyranoside (Sigma-Aldrich) diluted in 10 mM tris-HCl (pH 8.0), 150 mM NaCl, 5 mM EDTA, and 0.1% (v/v) Complete Protease Inhibitor Cocktail (Roche), with vortexing every 30 min. Lysates were cleared by centrifugation, and pHLA-I complexes were isolated by immunoaffinity purification using W6/32 mAb (RRID:AB_1107730) crosslinked to protein A beads (GE Healthcare) with dimethyl pimelimidate (Sigma-Aldrich). Beads were loaded into GELoader Tips (Thermo Fisher Scientific) and washed and pHLA-I complexes eluted with 10% (v/v) acetic acid. The eluates were further concentrated by SpeedVac, acidified with 10% trifluoroacetic acid (TFA) and loaded into prewashed and preequilibrated C18 stage tips (Thermo Fisher Scientific). After further washing, peptides were eluted with 50% (v/v) acetonitrile (ACN) and 0.05% (v/v) TFA. Samples were dried, resuspended in 0.05% TFA and 2% ACN, and spiked with CMV pp65_495–503_ peptide (10 fmol/μl; NLVPMVATV) as an internal control.

Using a Dionex UltiMate 3000 RSLCnano system, peptides were loaded onto a C18 Acclaim PepMap nanocolumn (5 mm by 300 μm; Thermo Fisher Scientific) and separated with an Acclaim PepMap C18 nanocolumn (50 cm by 75 μm; Dionex) coupled to a nano-electrospray ionization Q Exactive HF mass spectrometer (Thermo Fisher Scientific). Separation was achieved with a linear gradient of 2 to 90% buffer B (80% ACN and 0.05% formic acid) at a flow rate of 220 nl/min over 90 min at 35° to 45°C.

Full MS spectra were acquired from 375 to 1500 mass/charge ratio, at a resolution of 60,000 with an automatic gain control (AGC) target of 3,000,000. Precursors were selected using Top20 mode at a resolution of 15,000 with an AGC target of 50,000, a maximum injection time of 120 ms, and a dynamic exclusion of 20 to 30 s. Unassigned and precursor ion charge states of 1 or >5 (or >4 in some cases) were excluded. The peptide match option was set to “preferred.”

Data were analyzed using PEAKS 8.1 (Bioinformatics Solutions). Sequence interpretation was carried out using no enzyme specification, a mass tolerance precursor of 10 parts per million for peptides, and 0.05 for fragment ions. Sequence matching was done against a database containing (i) all human SwissProt entries (downloaded November 2017); (ii) amino acid sequences translated from RNA sequencing of the CVB1 and CVB3 strains used; (iii) an in-house database compiled as described (*16*), containing 679,576 and 530,424 predicted peptide splice products composed of major known and candidate β cell antigen sequences with CVB1 and CVB3 sequences, respectively; (iv) large T antigen from simian virus (UniProt P03070) used for β cell immortalization; and (v) putative polyproteins from a xenotropic murine leukemia virus (UniProt F8UU35, F8UU37) known to be present in ECN90 β cells (*48*). Posttranslational modifications detected with the PEAKS PTM built-in module were disregarded as likely artifactual, and the unmodified amino acid sequence was retained. A false discovery rate of 5% was set using a parallel decoy database search. Only the peptides with an 8– to 12–amino acid length compatible with HLA-I binding were retained.

### In silico selection of candidate epitopes

A parallel in silico search for nonamer peptides predicted to bind HLA-A2 or HLA-A3 was performed for all six CVB strains, using the following reference sequences: CVB1 AAC00531.1 (UniProt P08291), CVB2 AOW42548.1 (UniProt A0A1D8QMF3), CVB3 AFC88096.1 (UniProt H9B4F8), CVB4 AHB37371.1 (UniProt W8DN51), CVB5 AFO42818.1 (UniProt I7AVS5), and CVB6 AAF12719.1 (UniProt Q9QL88). The amino acid positions of each peptide are reported with reference to the first serotype listed. Criteria used for this search were as follows: a binding affinity (half-maximal inhibitory concentration) <40 nM, as predicted by both NetMHCpan 4.0 through the IEDB HLA-I prediction interface and NetMHC 4.0; or an identity of ≥3 consecutive amino acids with a β cell antigen sequence (INS, GAD, IA-2, ZnT8, IAPP, and IGRP) in central TCR contact residues (positions P3-P7). Peptides containing cysteines or found only in CVB2 or CVB6 were excluded. Two CVB peptides previously reported (*49*) were appended to this list: CVB1-2-3-4-5-6_1502–1510_ (GIIYIIYKL, IEDB #20311) and CVB2–4–5-6_1589-1598_ (ILMNDQEVGV, IEDB #27205).

### HLA-I binding assays

Peptides predicted to bind HLA-A2 or HLA-A3 (NetMHCStabPan 1.0) were synthesized (>95% pure, Synpeptide), and binding was tested using biotin-tagged HLA-A2 and HLA-A3 monomers (ImmunAware), as described (*19*). Briefly, biotinylated monomers were folded (1.2 nM final concentration) and captured on streptavidin-coated beads (6 to 8 μm, Spherotech), followed by incubation with anti–β2-microglobulin BBM.1 mAb (RRID:AB_626748) and then with AF488-labeled goat polyclonal anti-mouse IgG (RRID:AB_2728715). Positive controls included the HLA-A2–binding peptides Flu MP_58–66_ (GILGFVFTL) and TYR_369–377_ (YMDGTMSQV) and the HLA-A3–binding peptide Flu NP_265–273_ (ILRGSVAHK). The peptide CHGA_382–390_ (HPVGEADYF) was used as negative control for both HLA-A2 and HLA-A3. Beads were acquired on a BD LSRFortessa cytometer and analyzed by gating on single beads and AF488-positive events. Results are expressed as relative fluorescence intensities (RFIs), i.e., the median fluorescence intensity fold increase of the tested pHLA-I complex compared with the negative control. For HLA-A2, peptides with RFI > 6 were considered as intermediate binders and RFI > 20 as strong binders. For HLA-A3, peptides with RFI > 15 were scored as intermediate binders and RFI > 40 as strong binders.

### Study participants, specimens, HLA typing, and CVB serology

Study participants (table S1) were recruited in Paris and Miami and gave written informed consent under ethics approval DC-2015-2536 Ile-de-France I and University of Miami IRB protocol 1995-119, respectively. HLA-A2 (A*02:01) and HLA-A3 (A*03:01) typing was performed with AmbiSolv primers (Dynal/Thermo Fisher Scientific) and by DKMS (Tübingen, Germany) in case of ambiguities. Blood was drawn into 9-ml sodium heparin tubes, and PBMCs were isolated and frozen/thawed as described (*16*, *50*). Frozen/thawed samples were used throughout the study. Neutralizing antibody titers against the six CVB serotypes were measured with a plaque neutralization assay as described (*51*). Frozen PLN, spleen, and blood cells, provided by nPOD and the Human Atlas of Neonatal Development and Early Life Immunity program (HANDEL-I; table S3), were thawed in the presence of benzonase (50 U/ml; Merck 70664-3) and mifepristone (100 nM; Invitrogen H110-01).

### Combinatorial HLA-I MMr assays

HLA-A2 and HLA-A3 MMrs (immunAware) were produced and used as described (*16*, *19*, *20*). pHLA complexes were conjugated with fluorochrome-labeled streptavidins (1:4 ratio) and used at a final concentration of 8 to 27 nM, modulated to correct for the variable staining index of each streptavidin and thus visualize a distinct double-MMr^+^ population for each fluorochrome pair. PBMCs were thawed in prewarmed AIM-V medium (Thermo Fisher Scientific), washed, counted, and incubated with 50 nM dasatinib for 30 min at 37°C before negative magnetic CD8^+^ T cell enrichment (RRID:AB_2728716). Staining with combinatorial double-coded MMr panels was performed for 20 min at 20°C in 20 μl of PBS-dasatinib for 10^7^ cells followed, without washing, by staining at 4°C for 20 min with mAbs CD3-APC-H7 (RRID:AB_1645475), CD8-PE-Cy7 (RRID:AB_396852), CD45RA-FITC (RRID:AB_395879), CCR7-BV421 (RRID:AB_2728119), CD25-BV510 (RRID:AB_2629671), PD-1-BV605 (RRID:AB_2563212), and Live/Dead Aqua (Thermo Fisher Scientific). After washing, cells were acquired on an LSRFortessa configured as described (*16*) and analyzed with FlowJo v10 and GraphPad Prism 9. Candidate epitopes that did not yield any appreciable MMr staining or fluorochrome pairs not coding for a pHLA MMr provided negative controls for each panel. Positive control peptides included in the MMr panels were HCV PP_1406–1415_ (naïve viral control; KLVALGINAV; IEDB #32208) and/or MelanA_26–35_ ELA (naïve self-control; ELAGIGILTV; IEDB #12941) and influenza virus (Flu) MP_58–66_ (recall viral control; GILGFVFTL; IEDB #20354) for HLA-A2; and HIV Nef_84–92_ (naïve viral control; AVDLSHFLK; IEDB #5295) and influenza virus NP_265–273_ (recall viral control; ILRGSVAHK; IEDB #27283) for HLA-A3.

For the detection of CVB1_1132–1140_/GAD_272–280_ cross-reactive CD8^+^ T cells, the CVB1_1132–1140_ MMr pair (PE-CF594 and BV711) shared the BV711 fluorochrome with the GAD_272–280_ MMr pair (PE and BV711), thus allowing selective gating of T cells recognizing only CVB1_1132–1140_ or GAD_272–280_ (i.e., double MMr^+^) or both (i.e., triple MMr^+^), as described (*20*).

### In situ MMr staining on spleen, PLN, and pancreas tissue sections

Frozen tissue sections of the spleen, PLN, and different regions of the pancreas (head, body, and/or tail) were from nPOD nondiabetic (*n* = 5), double-aAb^+^ (*n* = 2), and T1D (*n* = 8) cadaveric organ donors (table S4; Ethics Committee #215/17S at Technical University of Munich and Helmholtz Munich Institute of Diabetes Research). For validating in situ staining with APC-labeled MMrs, PBMCs were isolated from HLA-A2^+^ and HLA-A3^+^ healthy donors using Lymphoprep density gradient centrifugation and stored at −80°C till use. Frozen/thawed PBMCs were magnetically depleted of CD8^−^ cells by magnetic-activated cell sorting–positive magnetic selection (Miltenyi). Isolated CD8^+^ cells were then incubated for 20 min with the indicated MMrs (10 nM) diluted in 2% goat serum in PBS and fixed with 4% Image-iT Fixative Solution (Thermo Fisher Scientific) for 15 min. CD8^+^ cells were then cytospun on a microscope slide and blocked with 10% goat serum and Human TruStain FcX (RRID:AB_2818986; 1:20) for 20 min at 4°C. MMr signal amplification was obtained with a mouse anti-APC mAb (RRID:AB_345357; 1:1000) for 1 hour at room temperature (RT). An AF647-conjugated goat anti-mouse IgG1 Ab (RRID:AB_141658; 1:800) was then added together with a rabbit anti-human CD3 Ab (RRID:AB_2335677; 1:300) for 1 hour at RT. CD8^+^ cells were detected by adding an AF750-conjugated goat anti-rabbit IgG Ab (RRID:AB_1500687; 1:800). Sections were counterstained with Hoechst 33342 (Invitrogen; 1:5000) for 8 min and mounted with ProLong Gold Antifade (Invitrogen). Sections were then scanned using an Axio Scan.Z1 slide scanner (Zeiss) and a 20×/0.8 numerical aperture Plan-Apochromat (*a* = 0.55 mm) objective.

Frozen tissue sections were prefixed with 0.2% Image-iT Fixative Solution for 5 min at 4°C. After gentle washing, sections were incubated with MMrs (10 nM) diluted as above for 3 hours at 4°C, washed, fixed with 4% Image-iT for 15 min, blocked with 10% goat serum and Human TruStain FcX as above, and incubated with mouse anti-APC mAb (1:50) for 1 hour at RT. AF647-conjugated goat anti-mouse IgG1 (1:800) was then added for 1 hour at RT together with rabbit anti-human CD8 Ab (RRID:AB_304247; 1:150), followed by AF750-conjugated goat anti-rabbit IgG (1:200). Hoechst counterstaining, mounting, and scanning were performed as above.

For image analysis, a mean of 23 regions of interest (ROIs) per donor from the spleen and whole slide images for PLNs were analyzed for each section. Briefly, the total number of cells per ROI or tissue area was determined using QuPath (*52*) with Stardist, a deep learning–based method of two-dimensional nucleus detection (*53*). The presence of MMr^+^CD8^+^ T cells was assessed manually. Cell density values were obtained by calculating the number of positive cells divided by the area (in square millimeters). Pancreatic tissue sections were first assessed for the presence of insulitis. Because of the scarcity of islet-infiltrating immune cells and MMr^+^ cells, further quantitative assessment was not performed.

VP1 immunofluorescence staining was performed on pancreas tissue sections from three T1D donors: nPOD 6070 (RRID:SAMN15879127; female, 23 years old; T1D duration, 7 years), nPOD 6228 (RRID:SAMN15879284; male, 13 years old; T1D duration, 0 years), and nPOD 6243 (RRID:SAMN15879299; male, 13 years old; T1D duration, 5 years). Four-micrometer sections were dewaxed in Histoclear, rehydrated in degrading ethanol concentrations (100, 95, and 70%) before heat-induced epitope retrieval [10 mM citrate (pH 6)]. Sections were blocked with 5% goat serum and incubated with mouse anti-VP1 Ab 5-D8/1 (RRID:AB_2118128; 1:1500) for 1 hour at RT. AF488-conjugated goat anti-mouse IgG Ab (RRID:AB_2534069; 1:400) was then added for 1 hour at RT. This was followed by incubation with guinea pig anti-insulin Ab (RRID:AB_2617169; 1:600) for 1 hour at RT. AF555-conjugated goat anti-guinea pig IgG Ab (RRID:AB_2535856; 1:400) was then added for 1 hour at RT along with 4′,6-diamidino-2-phenylindole (1 μg/ml). Sections were mounted and scanned using the Vectra Polaris slide scanner (Akoya Biosciences). Quantification was performed using the Indica HALO image analysis platform. Manual annotation around the cell periphery was performed for >50 VP1^+^ and VP1^−^ β cells to measure cell area, perimeter, and diameter.

### In vitro T cell stimulation, TCR sequencing, and reexpression

To generate PLN T cell lines, frozen/thawed single-cell suspensions were bulk-sorted by flow cytometry for CD4^+^ and CD8^+^ T cells. CD8^+^ T cells (10^5^ per well, four wells each) were stimulated with anti-CD28 (5 μg/ml) and irradiated autologous spleen-derived EBV-immortalized B cells pulsed with the indicated peptides (50 μg/ml), with interleukin-2 (IL-2; 20 U/ml) and IL-15 (10 ng/ml) added on day 6. On day 14, cultures were split and recalled with the stimulating peptide (50 μg/ml), the negative control peptide WNV_3098–3106_, or dimethyl sulfoxide diluent pulsed on irradiated autologous B cells with anti-CD28. After 48 hours, supernatants were collected and assayed for cytokine secretion by cytokine bead array (BD).

Single-cell TCR sequencing was performed on frozen/thawed cells rested overnight, followed by CD8^+^ magnetic enrichment and MMr staining, sorting in 96-well plates with a BD FACSAria II and a multiplex nested polymerase chain reaction, as described (*18*). Sequences were assigned using the international ImMunoGeneTics information system (www.imgt.org) and visualized as heatmaps using R software (www.github.com/raivokolde/pheatmap).

5KC T-hybridoma TCR transductants were generated as described (*22*). Briefly, 5KC cells were transduced with the NFAT-driven fluorescent reporter ZsGreen-1 along with human CD8 by spinoculation with the retroviral supernatant produced from Phoenix-ECO cells (ATCC CRL-3214). These cells were subsequently transduced with retroviral vectors encoding a murine TRAC chimeric TCRα gene followed by a porcine Teschovirus-1 2A peptide and a murine TRBC chimeric TCRβ gene (Twist Bioscience).

Primary CD8^+^ T cell transductants were generated from CD8^+^ T cells magnetically enriched from fresh PBMCs and plated at 20,000 cells per well. After activation with anti-CD3 and anti-CD28 microbeads (Thermo Fisher Scientific) for 48 hours in AIM-V medium, cells were transduced with lentiviral vectors encoding the above chimeric TCR construct and supplemented with IL-2 (40 U/ml; Proleukin, Clinigen).

To validate antigen specificity, transductants (20,000 cells per well in 96-well U-bottom plates) were stimulated for 18 hours with individual peptides added at 10-fold serial dilutions from 100 μM to 100 fM in the presence of K562 cells (50,000 cells per well) transduced with an HLA-A2 or HLA-A3 monochain construct fused with β2-microglobulin, followed by analysis of ZsGreen-1 expression (for 5KC transductants) or intracellular staining for macrophage inflammatory protein-1β (RRID:AB_357303), tumor necrosis factor–α (RRID:AB_398566), IL-2 (RRID:AB_2573518), and IFN-γ (RRID:AB_1272026) for primary CD8^+^ transductants.

### In vitro recall and cytotoxicity assays on CVB-infected β cells

For recall assays on 5KC TCR transductants, the HLA-A2/A3^+^ ECN90 β cell line was plated 24 hours ahead at 5 × 10^4^ per well in a 96-well flat-bottom Matrigel-coated plate in BSA-free DMEM/F12 Advanced medium supplemented as above. It was then pulsed for 1 hour with serial concentrations of CVB1_1356–1364_/CVB3_1359–1367_ peptide or left unpulsed, followed by CVB3 infection (300 MOI) for 1 hour, gentle washing, and addition of transductants. After 18 hours, cells were collected, stained with Live/Dead Red, and fixed with the eBioscience Foxp3/Transcription Factor Staining Buffer Set. NFAT-driven ZsGreen fluorescence was then acquired on a BD LSRFortessa after gating on viable single cells.

For CVB-eGFP infection assays, ECN90 β cells were seeded at 1.5 × 10^4^ per well (~40% confluency) in precoated 96-well plates and incubated overnight. Following infection with CVB-eGFP (*46*) at different MOIs for 1 hour at 37°C, Incucyte Cytotox Red (Essen Bioscience #ESS4632; 1:4000) was added for counting dead cells. Real-time image acquisition was performed on an Incucyte S3 (Sartorius) at four images per well and one scan/hour for 48 hours. Images were processed with the Incucyte software by defining masks on the green (CVB-eGFP^+^) and red (dead) β cell areas.

For T cell cytotoxicity assays, ECN90 β cells were stained with 1 μM CellTracker Red CMTPx dye (Thermo Fisher Scientific #C34552) for 30 min in BSA-free medium and seeded as above. After 6 hours, they were infected with CVB3 at different MOIs for 1 hour at 37°C or loaded with 1 μM cognate CVB peptide as positive control. Following addition of primary CD8^+^ T cell transductants at different T:β cell ratios, Incucyte Cytotox Green (Essen Bioscience #ESS4633; 1:4000) was added for counting dead cells. Real-time image acquisition and processing were performed as above by defining single-cell and cluster masks on the green (dead cell) and red (β cell) area. β cell death was defined as the percent green/red area out of total red area in each well after subtraction of the 8 hours background signal.

### IFN measurements

IFN-α2a, IFN-β, IFN-γ, and IFN-λ concentrations in the supernatants (25 μl each) of CVB-infected and noninfected ECN90 β cells, cultured alone or with primary CD8^+^ T cell transductants, were measured by MSD U-PLEX assays (Meso Scale Discovery #K15094K-1).

### Statistical analysis

Data are shown as median (range) or mean ± SEM. Significance was assessed using two-tailed tests with a cutoff value of α = 0.05, as detailed for each figure.
