# Supplementary material for: Coxsackievirus infection induces direct pancreatic β cell killing but poor antiviral CD8+ T cell responses
Source: Sci Adv. 2024 Mar 6;10(10):eadl1122. doi: 10.1126/sciadv.adl1122 (PMC10917340; doi:10.1126/sciadv.adl1122)
Supplement: Supplementary file 1 — Figs. S1 to S9 Tables S1 to S4 Legends for movies S1 to S5 [file sciadv.adl1122_sm.pdf]

Science Advances
AAAS

Supplementary Materials for

Coxsackievirus infection induces direct pancreatic β cell killing but poor antiviral CD8$^+$ T cell responses

Federica Vecchio *et al.*

Corresponding author: Roberto Mallone, roberto.mallone@inserm.fr

Sci. Adv. **10**, eadl1122 (2024)
DOI: 10.1126/sciadv.adl1122

The PDF file includes:

Figs. S1 to S9
Tables S1 to S4
Legends for movies S1 to S5

Other Supplementary Material for this manuscript includes the following:

Movies S1 to S5

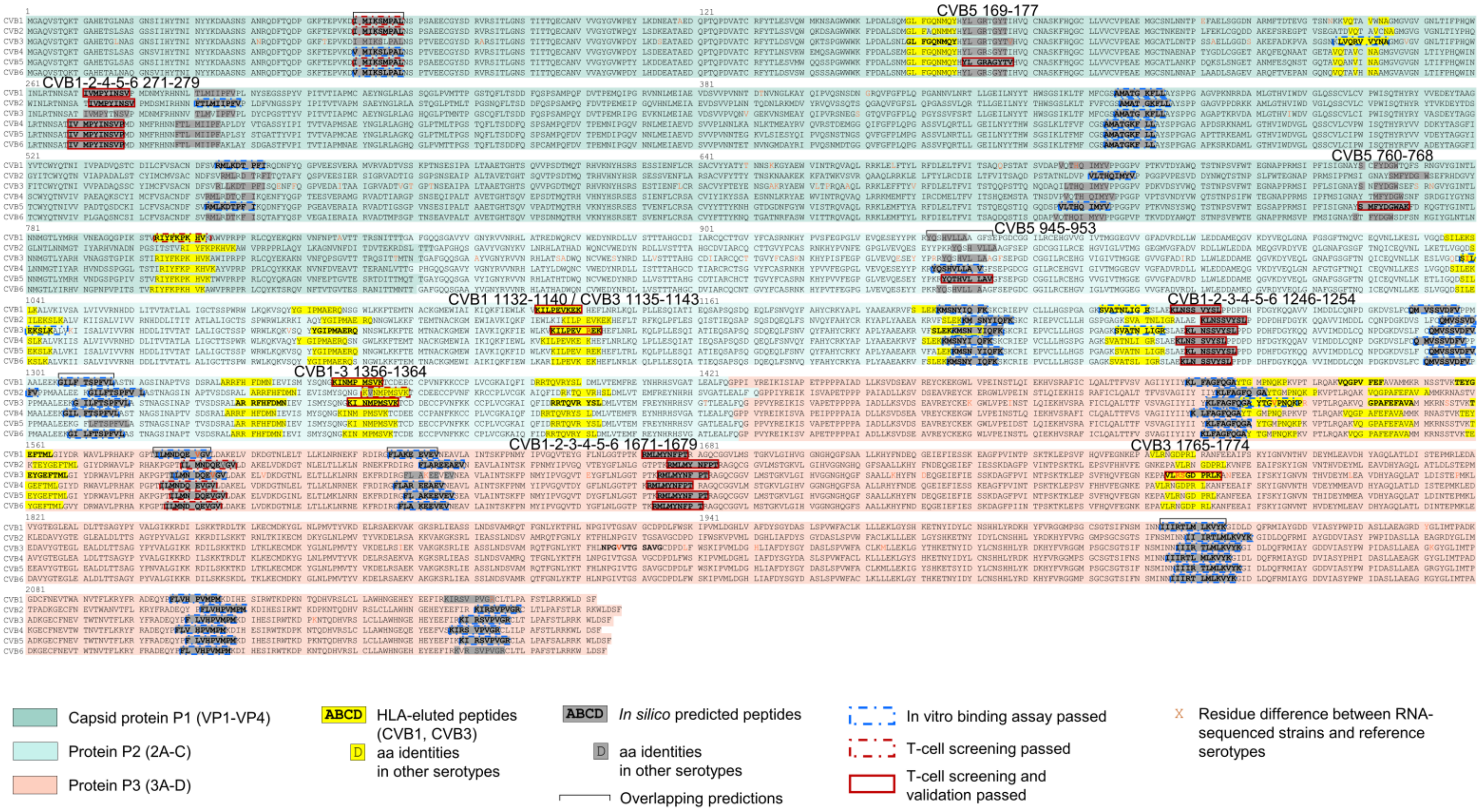

Fig. S1. Mapping of HLA-eluted and *in silico* predicted peptides and conservation across CVB serotypes. ECN90 β cells were infected with either CVB1 or CVB3. The pHLA-I complexes were purified and peptides sequenced by MS (bold fonts, highlighted in yellow). A parallel *in silico* search across relevant strains (see Materials and Methods) identified nonamer peptides predicted to bind HLA-A2 or HLA-A3 (bold font, highlighted in grey). Within these peptides, the aa conserved across serotypes are highlighted in yellow (for HLA-eluted peptides) or grey (for *in silico* predicted peptides). All the peptides were tested for their ability to bind to the corresponding HLA-I monomers (Fig. S2). Binding peptides thus identified (encircled with blue dashes) were screened by combinatorial HLA-I multimer (MMr) assays. Those scored as MMr^+ in this screening phase are encircled with red dashes. Peptides whose T-cell recognition was confirmed in validation experiments (Fig. 3, Table S2) are encircled with continuous red lines, with their aa positions indicated on top. CVB1 and CVB3 aa sequences are those translated from the RNA-sequencing of strains used for *in-vitro* infection. The aa differences between the experimental (RNA-sequenced) strains used for HLA-I peptidomics and the reference sequences used for *in-silico* predictions are indicated in red letters.

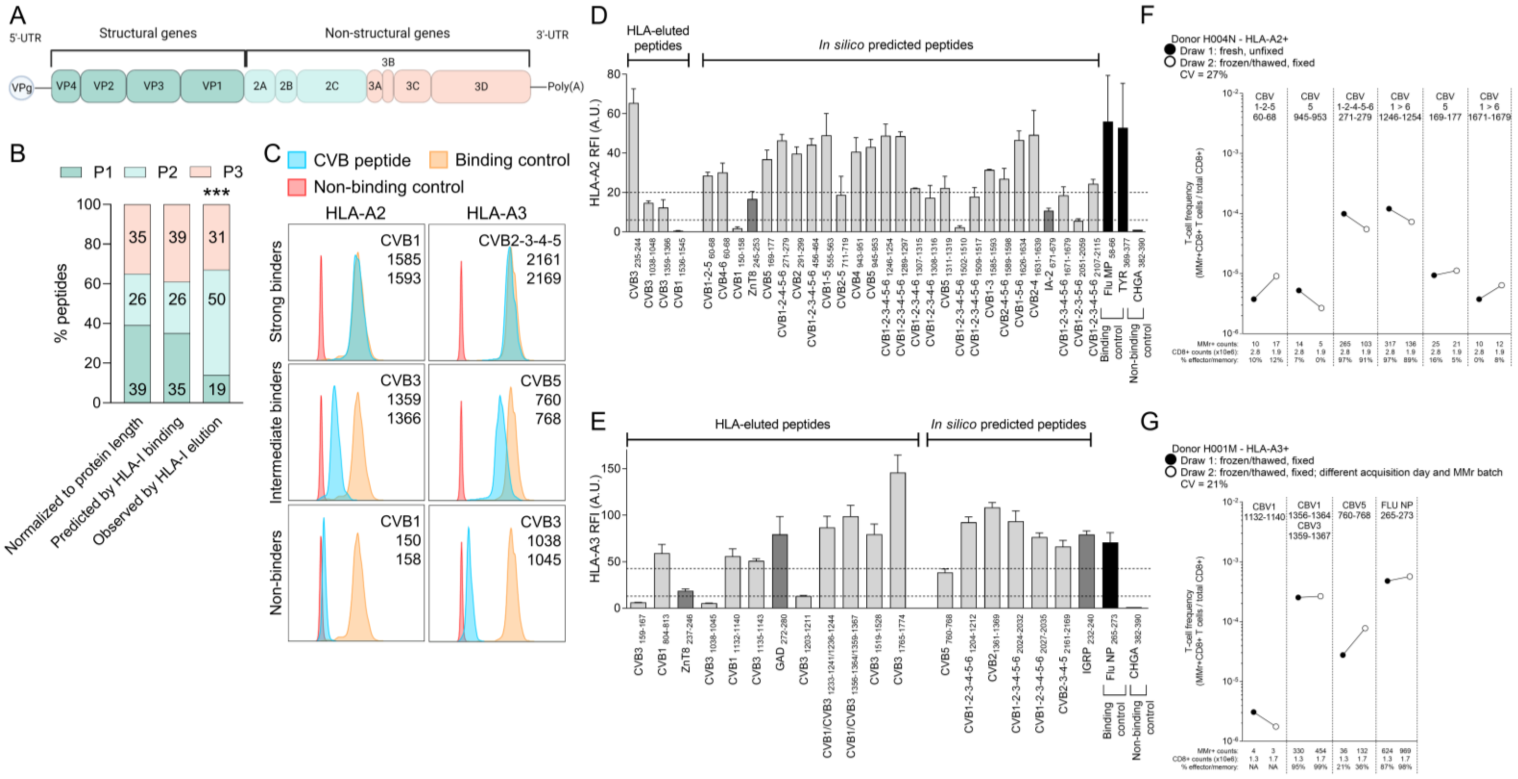

Fig. S2. Eluted vs. predicted HLA-I-binding CVB peptides, binding measurements and reproducibility of MMr assays. (**A**) Schematic of CVB genome. (**B**) Percent distribution of HLA-I-eluted unique peptides across the P1, P2 and P3 proteins (third bar) vs. those expected based on the aa length (first bar) and on the number of unique predicted binders of each protein (second bar; using NetMHCcons 1.1). Reference CVB1/CVB3 aa sequences were those translated from the RNA-sequencing of strains used for *in-vitro* infection. ****p*<0.0001 by χ^2 test. (**C**) HLA-I binding measurements. Biotinylated recombinant HLA-A2/A3 molecules were folded with peptides and β2-microglobulin, captured on streptavidin-coated beads and stained for β2-microglobulin. Representative staining for HLA-A2 (left) and HLA-A3 complexes (right) folded with CVB peptides (blue profiles) displaying strong, intermediate and no binding. Binding controls are Flu $MP_{58\text{-}66}$ and $TYR_{369\text{-}377}$ for HLA-A2 and Flu $NP_{265\text{-}273}$ for HLA-A3; non-binding control is $CHGA_{382\text{-}390}$. (**D-E**) Peptides were tested for their binding to HLA-A2 (D) and HLA-A3 (E). Horizontal dotted lines indicate cut-off for scoring strong, intermediate and non-binders. Bars represent mean ± SEM from 2-3 different experiments of the relative fluorescence intensity (RFI; median FI arbitrary units (A.U.) normalized to the $CHGA_{382\text{-}390}$ non-binding peptide). *In-silico* predicted HLA-A2-binding peptides $CVB1_{150\text{-}158}$ (KLPDALSQM; and its homologous $ZnT8_{245\text{-}253}$, VMGDALGSV), CVB1-2-3-4-5-$6_{1502\text{-}1510}$ (GIIYIIYKL; IEDB#20311) and CVB1-2-3-5-$6_{2051\text{-}2059}$ (VIASYPWPI) did not confirm as binders and are therefore not listed in Fig. 1G. (**F**) Reproducibility of CVB peptide-loaded HLA-A2 MMr assays. Donor H004N was analyzed using two separate blood draws and the same MMr batch, with PBMCs stained freshly after isolation and not fixed (black circles) or stained on frozen/thawed samples fixed after staining (white circles). Coefficient of variation (CV) of measured MMr^+CD8^+ T-cell frequencies was 27%. (**G**) Reproducibility of CVB peptide-loaded HLA-A3 MMr assays. Donor H001M was analyzed using frozen/thawed PBMCs from two separate blood draws and MMr batches, with PBMCs fixed after staining. CV=21%. MMr^+ counts, the number of total $CD8^+$ T cells acquired and the percent effector/memory fraction of MMr^+ cells are indicated. NA, not applicable (effector/memory fraction not assigned when <5 MMr^+ cells detected).

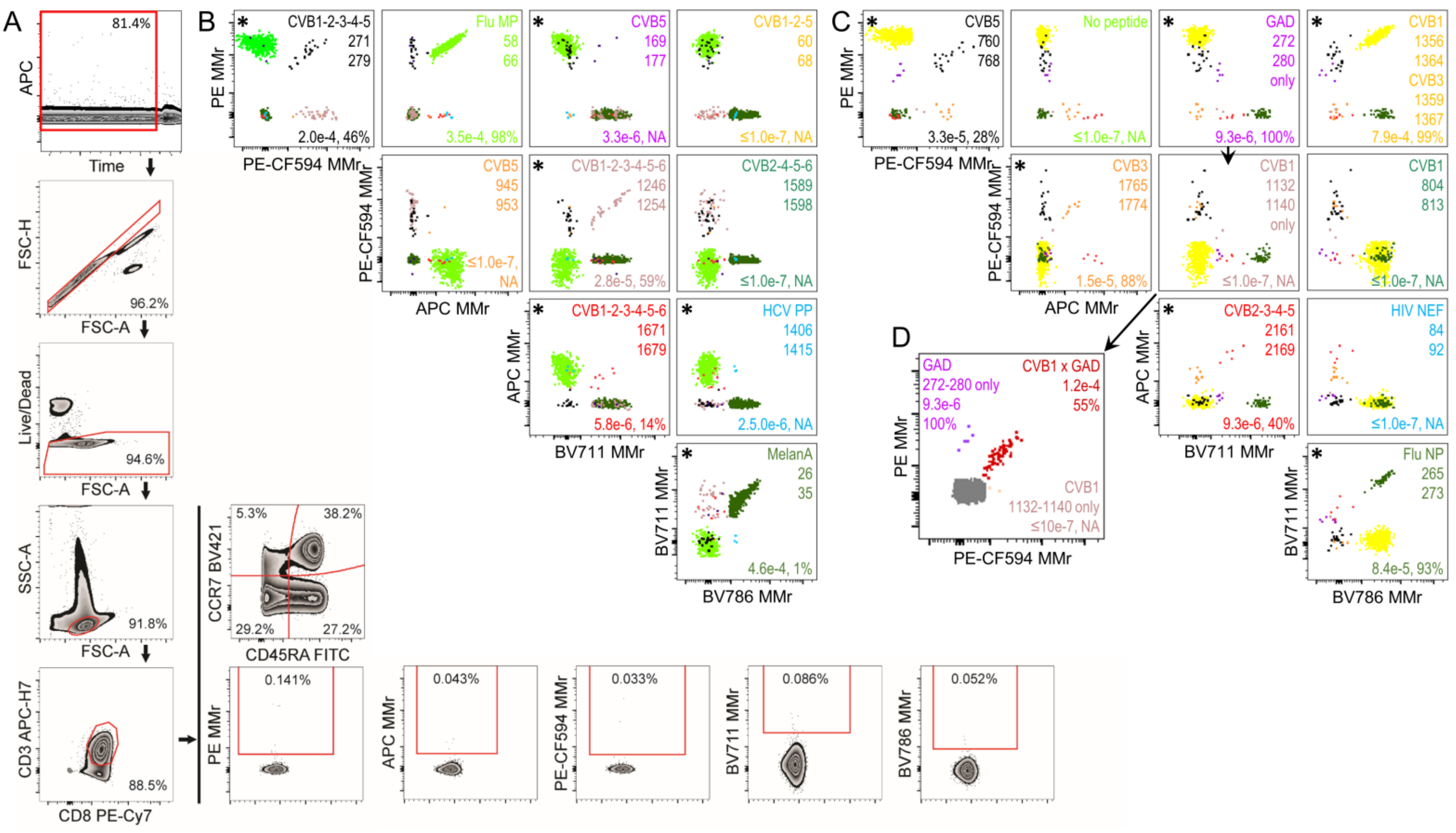

Fig. S3. Representative staining with the HLA-A2 and HLA-A3 MMr panels used for T-cell validation experiments. Frozen/thawed PBMCs from donor H004N (HLA-A2/A3$^+$) were magnetically depleted of $CD8^-$ cells before staining, acquisition, and analysis. (**A**) Gating strategy for the HLA-A2 MMr panel. After verifying the stability of each fluorescence signal over the acquisition time (example shown for APC), cells were sequentially gated on singlets (FSC-A/FSC-H dot plot), live cells (Live/Dead Aqua$^-$), small lymphocytes (FSC-A/SSC-A dot plot), $CD3^+CD8^+$ T cells, and total PE$^+$, APC$^+$, PE-CF594$^+$, BV711$^+$, and BV786$^+$ MMr$^+$ cells. Using Boolean operators, these latter gates allowed to selective visualize each double-MMr$^+$ population by including only those events positive for the corresponding fluorochrome pair. The CD45RA/CCR7 staining distribution of total $CD3^+CD8^+$ T cells is also shown. (**B**) The final HLA-A2 MMr readout obtained is shown for the 10 peptides analyzed. Events corresponding to each epitope-reactive population are overlaid in different colors within each dot plot. Peptides scoring positive for this donor are marked with an asterisk. Numbers in each panel indicate the MMr$^+$CD8$^+$ T-cell frequency out of total CD8$^+$ T cells and the percent effector/memory fraction among MMr$^+$ cells (NA when not assigned, i.e. <5 MMr$^+$ cells counted). (**C**) The final HLA-A3 MMr readout obtained is shown for the 9 peptides analyzed (with the PE/APC fluorochrome pair left empty). Data representation is the same as in panel B. (**D**) The gating strategy based on positivity for 2 MMr fluorochromes precludes visualization of $CVB1_{1132\text{-}1140}$/$GAD_{272\text{-}280}$ cross-reactive CD8$^+$ T cells, since they are positive for 3 fluorochromes (PE, PE-CF594 and the shared BV711 fluorochrome). Hence, one further gating was performed for these fractions by selecting cells positive for only 2 fluorochromes (PE-CF594/BV711$^+$, PE$^-$, labeled as "$CVB1_{1132\text{-}1140}$ only"; or PE/BV711$^+$, PE-CF594$^-$, labeled as "$GAD_{272\text{-}280}$ only") or for all 3 (PE/PE-CF594/BV711$^+$, labeled as "CVB1 × GAD"). The resulting overlaid dot plot is displayed (with cells negative for all 3 fluorochromes depicted in grey), with most T cells (shown in red) cross-recognizing $CVB1_{1132\text{-}1140}$ and $GAD_{272\text{-}280}$ epitopes.

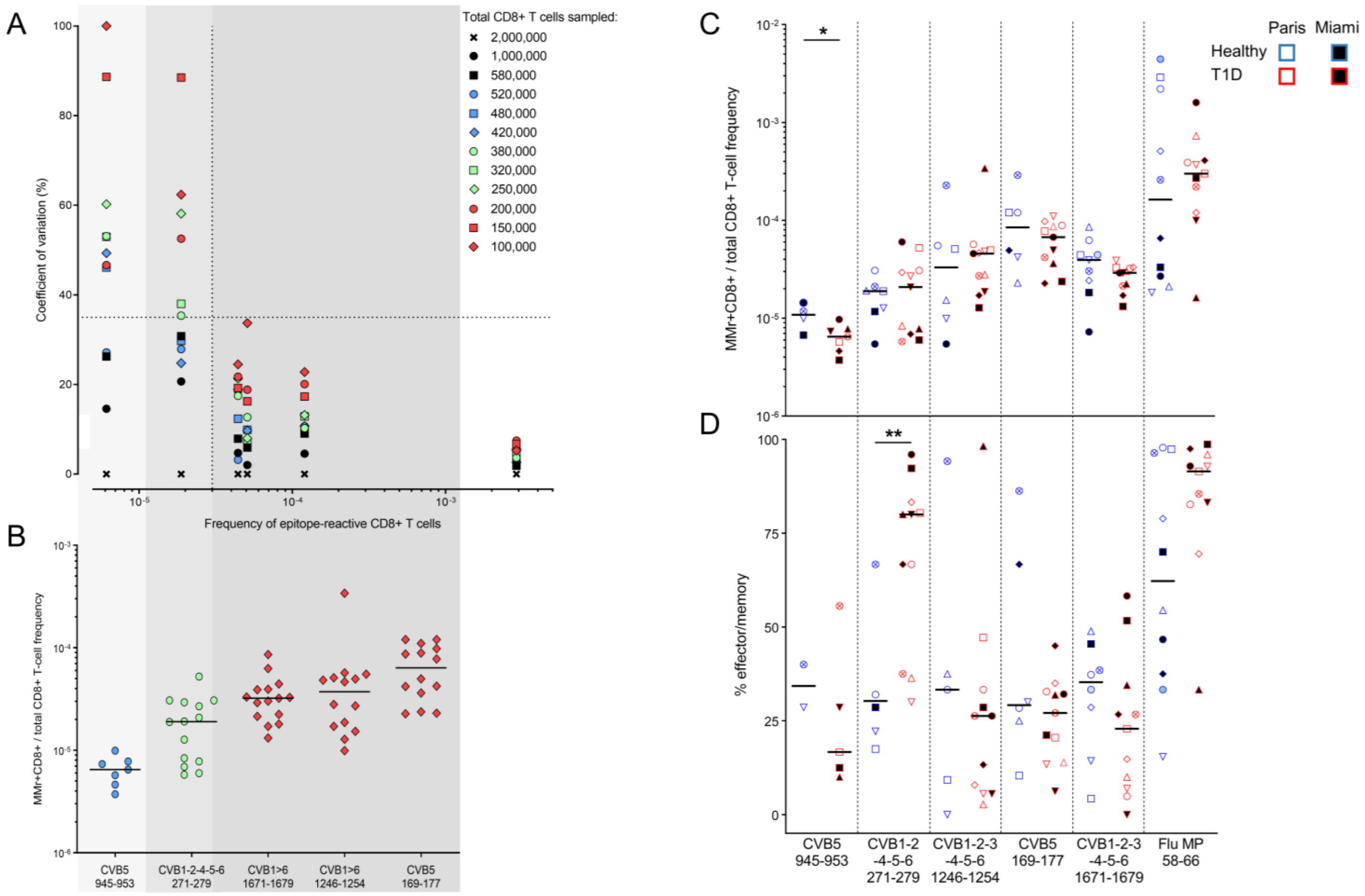

Fig. S4. Circulating HLA-A2-restricted CVB-reactive CD8$^+$ T cells in pediatric donors. (**A-B**) Minimal CD8$^+$ T-cell sampling size required to accurately measure CVB epitope-reactive CD8$^+$ T-cell frequencies. In panel A, decreasing numbers of total CD8$^+$ T cells from a single blood draw (donor H004N) were MMr-stained and acquired. The MMr$^+$CD8$^+$ T-cell frequency measured on the maximal number (2×10^6) of CD8$^+$ T cells acquired was considered as the gold standard (crossed symbols), and the CV of frequencies measured with lower T-cell numbers calculated accordingly. For CVB epitopes recognized by MMr$^+$CD8$^+$ T cells with a mean frequency >3/10^5 (vertical dotted line), the acquisition of ≥100,000 CD8$^+$ T cells (red diamonds) yields an accurate frequency measurement (CV<35%; horizontal dotted line). For MMr$^+$CD8$^+$ T-cell frequencies <2/10^5, ≥380,000 CD8$^+$ T cells (green circles) are required to maintain a CV<35%. For frequencies <7/10^6, ≥520,000 CD8$^+$ T cells (blue circles) are required to maintain a CV<35%. Panel B depicts the median frequency and distribution of MMr$^+$CD8$^+$ T cells using the indicated CVB peptides in pediatric donors with >500,000 CD8$^+$ T cells acquired. Based on these frequencies, the cut-offs defined in panel A for the minimal number of CD8$^+$ T cells acquired were applied for each peptide, as indicated by grey shades: ≥520,000 (blue circles) for CVB5$_{945\text{-}953}$, ≥380,000 (green circles) for CVB1-2-4-5-6$_{271\text{-}279}$, ≥100,000 (red diamonds) for all other epitopes. Using these cut-offs, only samples with accurate frequency measurements were retained for final analysis. (**C-D**) Frequency (C) and percent effector/memory phenotype (D) of MMr$^+$CD8$^+$ T cells recognizing the indicated CVB or control memory Flu MP$_{58\text{-}66}$ epitope in T1D (blue) and healthy (red) pediatric donors (Table S1). Data representation is the same as in Fig. 3; children recruited in Paris and Miami are represented with symbols filled in white and black, respectively. **p*=0.04 and ***p*=0.01 by Mann-Whitney U test. The HLA-A2 MMr staining panel was: CVB$_{1671\text{-}1679}$ PE/APC, CVB$_{169\text{-}177}$ PE/BV711, CVB$_{945\text{-}953}$ PE/BV786, CVB$_{1246\text{-}1254}$ APC/BV711, CVB$_{271\text{-}279}$ APC/BV786, Flu MP$_{58\text{-}66}$ BV711/BV786.

A

Spleen specimens (Fig. 4A-B)

MMr panel	Fluorochrome pair	Peptide
HLA-A2	PE / APC	HCV PP 1406-1415
	PE / BV711	CVB5 169-177
	PE / BV786	CVB5 945-953
	APC / BV711	CVB1-2-3-4-5-6 1246-1254
	APC / BV786	CVB1-2-4-5-6 271-279
	BV711 / BV786	Flu MP 58-66
HLA-A3	PE / APC	HIV nef 84-92
	PE / BV711	GAD 272-280*
	PE / BV786	CVB1 1356-1364 / CVB3 1359-1367
	APC / BV711	CVB1 1132-1140*
	APC / BV786	CVB5 760-768
	BV711 / BV786	Flu NP 265-273
HLA-A2 + HLA-A3	PE / APC	HLA-A3 HIV nef 84-92
	PE / BV711	HLA-A3 GAD 272-280
	PE / BV786	HLA-A3 CVB1 1356-1364 / CVB3 1359-1367
	APC / BV711	HLA-A2 CVB1-2-3-4-5-6 1246-1254
	APC / BV786	HLA-A2 CVB1-2-4-5-6 271-279
	BV711 / BV786	HLA-A3 Flu NP 265-273

PLN and PBMC specimens (Fig. 4C-D-E)

MMr panel	Fluorochrome pair	Peptide
HLA-A2	PE/BV650	CVB1-2-3-4-5-6 1246-1254
	APC/PE-CF594	CVB1 271-279
	BV711	Flu MP 58-66
HLA-A3	PE/BV650	CVB1 1356-1364 / CVB3 1359-1367
	APC/PE-CF594	CVB1 1132-1140
	BV711	Flu NP 265-273
HLA-A2 + HLA-A3	PE/BV650	HLA-A2 CVB1 271-279
	APC/PE-CF594	HLA-A3 CVB1 1356-1364 / CVB3 1359-1367
	BV711	HLA-A3 Flu NP 265-273
Phenotype markers	Far Red	Live/Dead
	APC-H7	CD3
	PE-Cy7	CD8
	FITC	CD45RA
	BV421	CCR7
	BV510	CD25
	BV605	PD-1

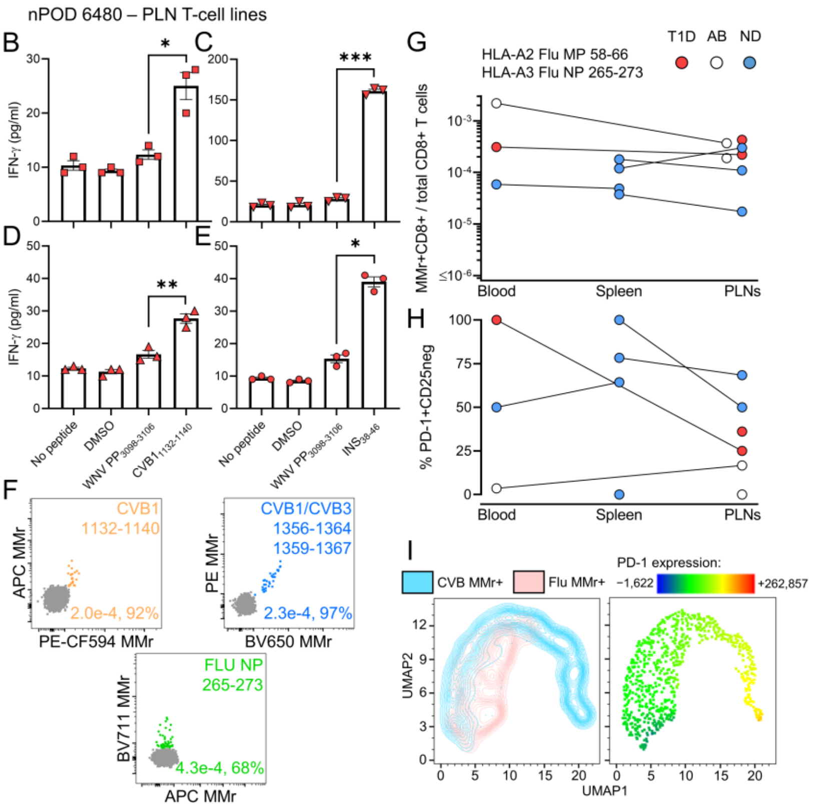

Fig. S5. CVB-reactive PLN T cells in HLA-A3$^+$ T1D nPOD case 6480, representative MMr staining on PLN cells and CVB *vs.* Influenza virus MMr$^+$CD8$^+$ T cells across tissues from nPOD donors. (**A**) MMr panels used for the analysis of nPOD spleen, PLN and PBMC specimens. The combined HLA-A2/HLA-A3 MMr panels were used for specimens from HLA-A2/A3$^+$ nPOD donors. The homologous CVB1$_{1132\text{-}1140}$/ GAD$_{272\text{-}280}$ peptides are marked with an asterisk. The MMr panel for PLN and PBMC specimens was also used for splenocytes from nPOD donors 6338, 6386, 6420, 6461 and 6480. The extended phenotypic panel used for PLN and PBMC specimens is also detailed. (**B-E**) PLN CD8$^+$ T cells were stimulated with the indicated CVB or INS$_{38\text{-}46}$ (INS$_{B14\text{-}22}$) peptides for 14 days, followed by a 48-h recall with the stimulating peptide, the negative control WNV PP$_{3098\text{-}3106}$ peptide or DMSO diluent and cytokine measurement on culture supernatants. Besides IFN-γ, TNF-α and IL-6 were also secreted in response to CVB1$_{1765\text{-}1774}$ peptide (not shown). Data is depicted as mean ± SEM of triplicate wells. **p*≤0.02, ***p*=0.003, ****p*=0.0007 by paired Student's t test. (**F**) Representative HLA-A3 MMr staining on PLN cells from T1D nPOD case 6362. Given the limited cell numbers available, frozen-thawed PLN cells were directly stained and acquired, without prior magnetic depletion of CD8$^-$ cells. The gating strategy is the same as in Fig. S3A, but each fluorochrome pair was here used for only one MMr, in order to allow sorting of individual CVB double-MMr$^+$ cells for TCR sequencing, with control Flu MMrs labeled with a single BV711 fluorochrome. The final readout obtained is shown for the 3 peptides analyzed. Each dot plot displays a color-coded overlay of individual double-MMr$^+$ subsets to visualize the separation of each epitope-reactive CD8$^+$ T-cell fraction relative to the MMr$^-$ population (gray). Numbers in each panel indicate the MMr$^+$CD8$^+$ T-cell frequency out of total CD8$^+$ T cells and the percent effector/memory fraction among MMr$^+$ cells. (**G-H**) Influenza virus MMr$^+$CD8$^+$ T cells in lymphoid tissues from nPOD cases (see details in Table S3). MMr$^+$CD8$^+$ T-cell frequencies (G) and percent PD-1$^+$CD25$^-$ MMr$^+$ cells (H; for donors/epitopes with cell counts ≥5) are depicted across available tissues. (**I**) FlowSOM uniform manifold approximation and projection (UMAP) analysis of CVB (CVB1$_{271\text{-}279}$ and CVB1$_{1356\text{-}1364}$) and Influenza virus (Flu MP$_{58\text{-}66}$ and Flu NP$_{265\text{-}273}$) MMr$^+$CD8$^+$ cells from all available tissues. Left, clustering of the two populations; right, the projected surface PD-1 expression.

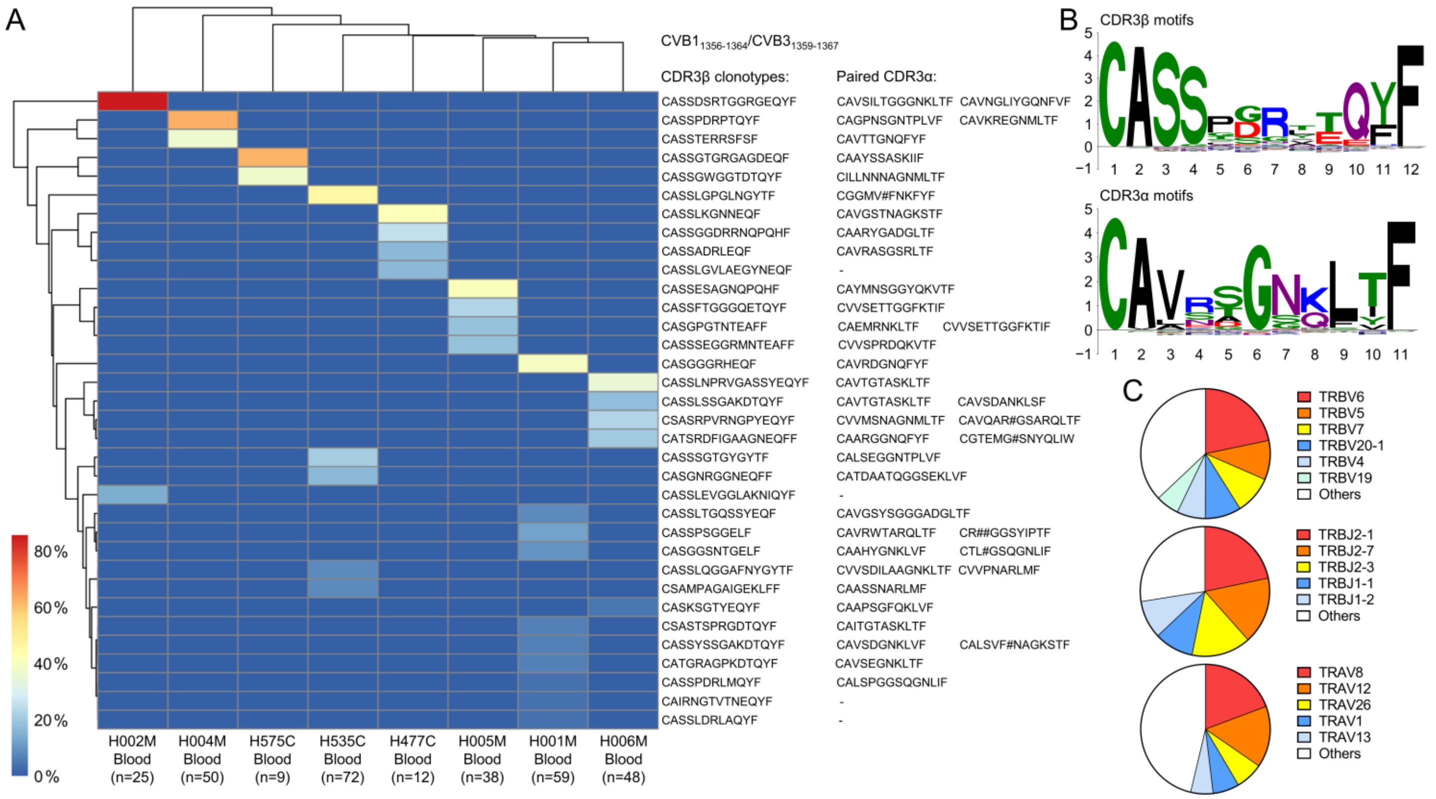

Fig. S6. Expanded TCR CDR3β clonotypes in individual CVB1/CVB3$_{1356\text{-}1364/1359\text{-}1367}$-reactive CD8$^+$ T cells from the blood of living CVB-seropositive healthy donors and common CDR3 motifs. (**A**) Expanded clonotypes were defined as those found in at least 2 MMr$^+$ cells in the same donor, and are clustered according to frequency (one columns per donor) and sequence similarity (one row per CDR3β clonotype). (**B**) Sequence logo plots displaying CDR3β (top) and CDR3α (bottom) motifs among all TCRs sequenced from CVB1/CVB3$_{1356\text{-}1364}$/$_{1359\text{-}1367}$ MMr$^+$CD8$^+$ T cells isolated from tissues and blood. The x-axis shows the CDR3 aa position after MMseqs2 centroid alignment. The y-axis shows the information content, with the size of each aa symbol proportional to its frequency. (**C**) Distribution of *TRBV* (top), *TRBJ* (middle) and *TRAV* (bottom) gene usage among 378 TCRs sequenced from CVB1/CVB3$_{1356\text{-}1364}$/$_{1359\text{-}1367}$ MMr$^+$CD8$^+$ T cells isolated from tissues and blood.

A

HLA-A2	CVB1-2-3-4-5-6 1246-1254<br>CVB1-2-4-5-6 271-279	KLNSSVYSL<br>IVMPYINSV
	CMV pp65 495-503<br>Flu MP 58-66	NLVPMVATV<br>GILGFVFTL
	WNV PP 430-438	SVGGVFTSV
HLA-A3	CVB1 1356-1364 / CVB3 1359-1367<br>CVB1 1132-1140	KINMPMSVK<br>KILPEVKEK
	CMV IE1 99-107<br>Flu NP 265-273	RIKEHMLKK<br>ILRGSVAHK
	WNV PP 3098-3106	LTYRHKVVK

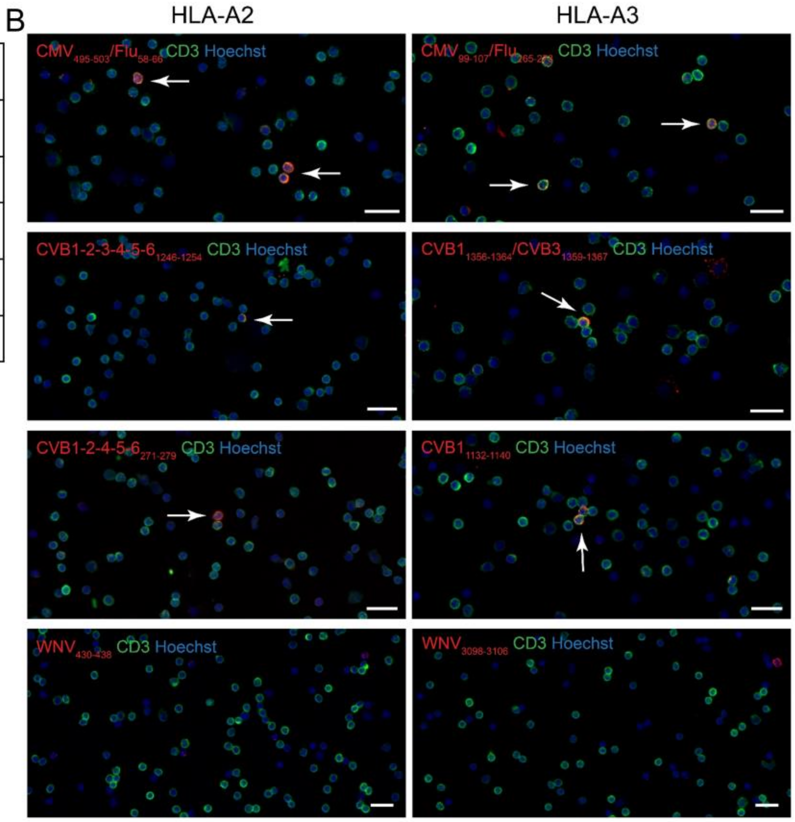

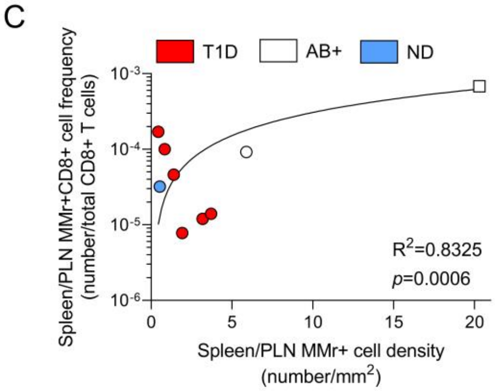

Fig. S7. Validation of *in-situ* MMr staining. (**A**) MMrs used for *in-situ* staining on human tissues. (**B**) Representative immunofluorescence images of isolated $CD8^+$ T cells cytospun on microscope glass slides. Cells were stained with the indicated pooled CMV/Flu, pooled CVB or single WNV peptide-loaded MMrs (red) and CD3 (green). Cell nuclei are stained in blue. White arrows point to MMr^+CD3^+ cells. Scale bars: 20 μm. (**C**) Correlation between CVB MMr^+CD8^+ cell frequencies measured by flow cytometry and CVB MMr^+CD8^+ cell densities quantified by tissue immunofluorescence. The samples analyzed by both techniques available for this comparison were all from spleen (round symbols), barring a PLN sample from double-aAb^+ case 6197 (square symbol).

A

	MOI 10 T:β 0.5:1	MOI 100 T:β 0.5:1	MOI 300 T:β 0.5:1	MOI 10 T:β 1:1	MOI 100 T:β 1:1	MOI 300 T:β 1:1	MOI 10 T:β 2:1	MOI 100 T:β 2:1	MOI 300 T:β 2:1
r	+0.93	+0.70	+0.97	+0.16	+0.92	+0.97	−0.75	−0.96	−0.85
95% CI	+0.78<br>+0.98	+0.26<br>+0.90	+0.91<br>+0.99	−0.41<br>+0.65	+0.76<br>+0.98	+0.93<br>+0.99	−0.92<br>−0.37	−0.99<br>−0.89	−0.95<br>−0.57
p	<0.0001	0.0067	<0.0001	0.5838	<0.0001	<0.0001	0.0024	<0.0001	0.0002

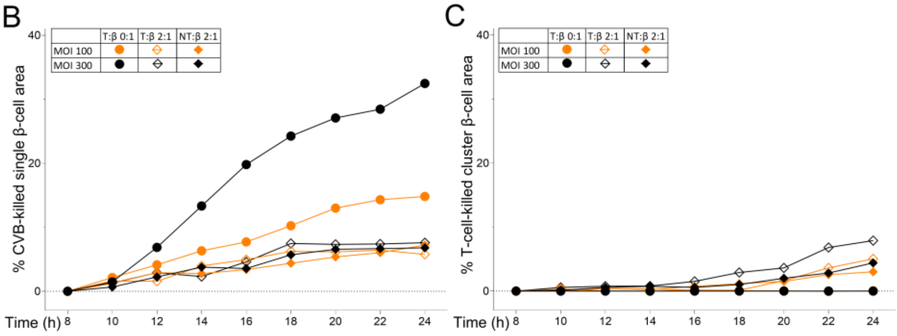

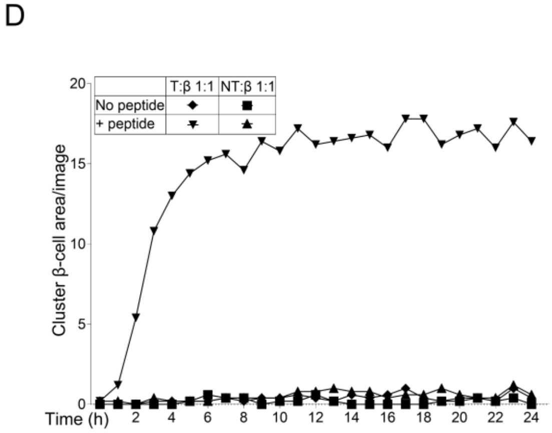

Fig. S8. Correlation between CVB-mediated and T-cell-mediated β-cell killing. (**A**) r, 95% confidence interval (CI) and *p* values of the correlation between CVB-mediated and T-cell-mediated β-cell killing depicted in Fig. 7D. (**B**) Equivalent inhibition of CVB-mediated single β-cell death by both transduced (T) and non-transduced (NT) $CD8^+$ T cells at a 2:1 T:β-cell ratio. (**C**) T-cell-mediated cluster β-cell death in the same conditions. (**D**) CVB peptide-specific β-cell killing by transduced (T) and non-transduced (NT) $CD8^+$ T cells. No killing is observed with non-transduced T cells.

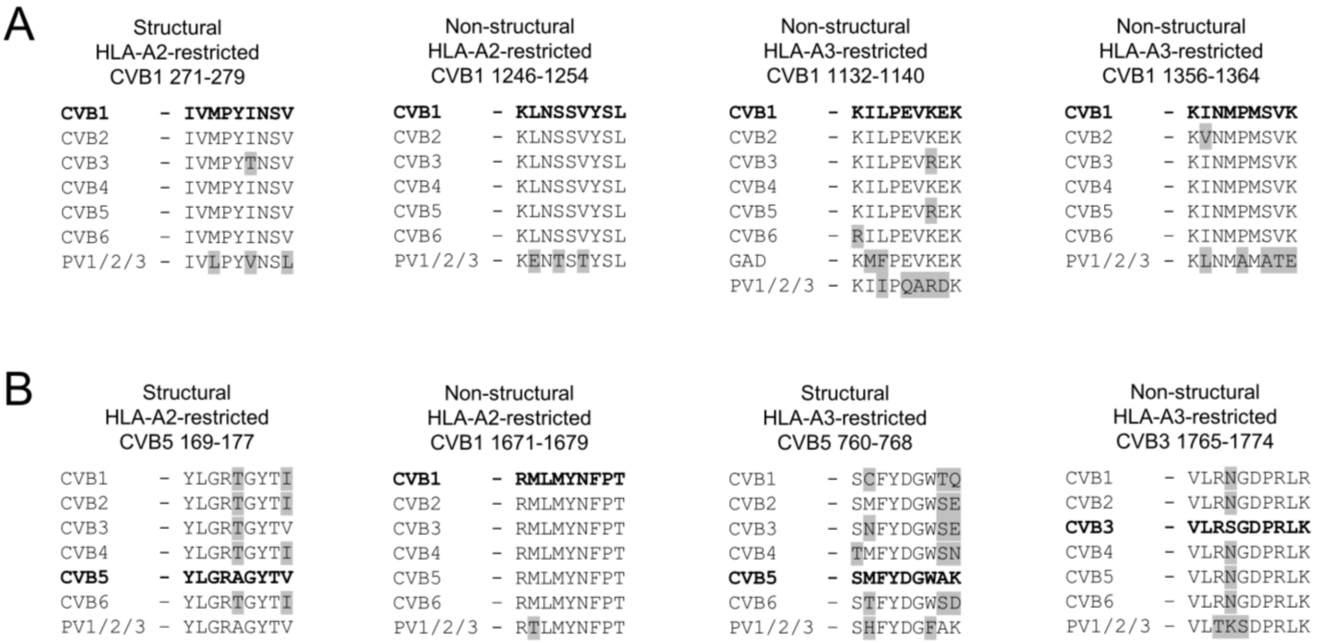

Fig. S9. Alignment of the main CD8+ T-cell epitopes identified across CVB and Poliovirus serotypes. Amino-acid differences are shaded in grey. For Poliovirus, the 3 serotypes included in the inactivated poliovirus vaccine were used as reference sequences: serotype 1 (Mahoney; GenBank V01149.1), 2 (MEF1; GenBank AY238473.1) and 3 (Saukett; GenBank KP247597.1). The alignment refers to the serotype with the closest match.

Study group	Code	Age (yrs)	Gender (M/F)	HLA	CVB1 NAbs	CVB2 NAbs	CVB3 NAbs	CVB4 NAbs	CVB5 NAbs	CVB6 NAbs	T1D duration (yrs)	Symbols
Screen (n=9)	H079O	38	F	A2	**≥16**	**≥16**	0	0	**≥16**	**4**	na	○
	H110S	60	F	A3	0	**≥16**	0	**≥16**	**≥16**	**4**	na	◇
	H112S	42	F	A3	**≥16**	**≥16**	0	**≥16**	**4**	**≥16**	na	□
	H158O	56	M	A2	**≥16**	**≥16**	**≥16**	**≥16**	**≥16**	**≥16**	na	□
	H170S	40	F	A2	**≥16**	**≥16**	**≥16**	**≥16**	**≥16**	0	na	▽
	H458C	33	M	A3	**≥16**	**≥16**	**≥16**	**≥16**	**≥16**	**4**	na	△
	H460C	24	M	A2	0	0	**≥16**	**≥16**	**≥16**	0	na	◍
	H463C	29	F	A2	**≥16**	**4**	**≥16**	0	**≥16**	**4**	na	▣
	H477C	34	F	A3	**4**	**≥16**	**≥16**	**≥16**	**≥16**	**4**	na	▽
Screen + Validation (n=6)	H004N	45	M	A2/A3	0	**≥16**	**≥16**	0	**≥16**	**4**	na	◇
	H106S	48	F	A2	**≥16**	**≥16**	**≥16**	**≥16**	**≥16**	**≥16**	na	◇
	H455C	25	F	A2	**≥16**	**≥16**	**≥16**	0	**4**	**≥16**	na	△
	H466C	25	F	A2	**≥16**	**≥16**	**≥16**	**≥16**	**≥16**	**≥16**	na	▽
	H478C	36	F	A3	0	**≥16**	0	**≥16**	**≥16**	0	na	◍
	H487C	39	M	A2	na	na	na	na	na	na	na	△
Validation (n=10)	H001M	27	M	A3	na	na	na	na	na	na	na	●
	H002M	36	M	A3	na	na	na	na	na	na	na	◆
	H003M	29	M	A3	na	na	na	na	na	na	na	■
	H004M	23	M	A3	na	na	na	na	na	na	na	▲
	H005M	28	M	A3	na	na	na	na	na	na	na	▼
	H006M	33	M	A3	na	na	na	na	na	na	na	⊗
	H372C	26	F	A2	0	**≥16**	**≥16**	**≥16**	0	**4**	na	⊗
	H423C	26	M	A3	na	na	na	na	na	na	na	○
	H459C	26	F	A3	**≥16**	**≥16**	**≥16**	0	**4**	**4**	na	▣
	H494C	56	F	A2/A3	na	na	na	na	na	na	na	⊗
T1D (n=11)	B001M	8	M	A2	**≥16**	**≥16**	0	**≥16**	0	**4**	3	◇
	B002M	12	M	A2	0	0	**≥16**	**≥16**	0	0	4	□
	B003M	12	M	A2	**4**	**≥16**	0	0	0	0	7	○
	B004M	19	M	A2	0	**4**	0	**≥16**	**≥16**	**4**	0.6	△
	B005M	16	M	A2	**4**	**≥16**	**≥16**	0	**≥16**	0	0.6	▽
	B006M	9	F	A2	**4**	**4**	0	**≥16**	**≥16**	**4**	6	⊗
	B091S	7	M	A2	na	na	na	na	na	na	0	●
	B241R	14	M	A2	**≥16**	0	**≥16**	0	0	**4**	0.02	◆
	B244V	17	M	A2	0	0	0	**≥16**	**≥16**	**4**	0	■
	B252R	11	M	A2	**≥16**	**≥16**	**≥16**	**≥16**	**≥16**	**≥16**	0	▲
	B234R	11	M	A2	0	**≥16**	**≥16**	**≥16**	**≥16**	**4**	0.02	▼
		**12 (7-19)**	**91% M / 9% F**		**60%**	**70%**	**50%**	**70%**	**60%**	**70%**	**0.6 (0-7)**	
Healthy (n=10)	HB001M	9	M	A2	na	na	na	na	na	na	na	◇
	HB002M	18	M	A2	**≥16**	0	**≥16**	**≥16**	0	**4**	na	□
	HB003M	13	F	A2	**≥16**	**≥16**	**≥16**	**≥16**	**4**	**4**	na	○
	HB004M	14	M	A2	**4**	**≥16**	0	**4**	**4**	**4**	na	△
	HB005M	11	M	A2	0	**≥16**	**≥16**	**≥16**	0	0	na	▽
	HB006M	9	F	A2	**4**	0	**≥16**	0	**≥16**	**4**	na	⊗
	HB007M	16	M	A2	**4**	0	**≥16**	0	**≥16**	**4**	na	◍
	HB030J	8	M	A2	**≥16**	**≥16**	**≥16**	**≥16**	**≥16**	**≥16**	na	●
	HB052J	13	M	A2	**4**	**≥16**	0	**≥16**	0	0	na	◆
	HB055J	13	M	A2	**≥16**	0	0	**≥16**	**≥16**	**≥16**	na	■
		**13 (8-18)**	**80% M / 20% F**		**89%**	**50%**	**60%**	**70%**	**60%**	**70%**		

Table S1. Donors recruited for T-cell experiments (related to Fig. 2, 3 and S4). Columns display study groups, donor code, demographics, HLA-A2/A3 typing, titers of neutralizing antibodies (NAbs) against CVB serotypes (positive titers of ≥16 in underlined bold, borderline titers of 4 in bold; corresponding to serum dilutions of 1/16 and 1/4, respectively; na, not available), T1D duration and the corresponding symbols used in Fig. 2, 3 and S4. HLA-A2^{+} donors recruited in Miami for T-cell validation experiments are indicated with black-filled symbols. T1D and healthy donors recruited in Miami are indicated with white- or blue-filled symbols. All other donors were recruited in Paris.

HLA	Strain	Position	Sequence	Pipeline	Screening round			Validation round	
					Frequency median	MMr+ counts median (range)	% effector/memory median (range)	Frequency median	% effector/memory median (range)
	CVB3	235-244	KLVQRVVYNA	MS	1.6 E-6	1 (0-3)	0 (0-0)		
	CVB3	1038-1048	SILEKSLKALV	MS	1.2 E-6	1 (0-2)	0 (0-100)		
	CVB1/CVB3	1359-1366	KINMPMSV	MS	1.0 E-7	0 (0-1)	0 (0-0)		
	CVB1-2-5	60-68	IMIKSMPAL	In silico	2.2 E-5	30 (8-155)	26 (0-70)	1.0 E-7	NA
	CVB4-6	60-68	VMIKSLPAL	In silico	5.3 E-6	2 (0-5)	20 (20-20)		
	**CVB5**	**169-177**	**YLGRAGYTV**	**In silico**	**5.4 E-5**	**14 (6-50)**	**10 (4-55)**	**7.3 E-6**	**14 (0-33)**
	**CVB1-2-4-5-6**	**271-279**	**IVMPYINSV**	**In silico**	**2.2 E-6**	**2 (0-24)**	**85 (0-100)**	**2.1 E-5**	**43 (26-82)**
	CVB2	291-299	FTLMIIPFV	In silico	1.5 E-6	1 (0-3)	0 (0-100)		
	CVB1-2-3-4-5-6	456-464	AMATGKFLL	In silico	1.0 E-7	0 (0-1)	0 (0-0)		
	CVB1-5	555-563	RMLKDTPFI	In silico	1.0 E-7	0 (0-1)	0 (0-0)		
HLA-A2 n=24 **n=5**	CVB2-5	711-719	VLTHQIMYV	In silico	1.0 E-7	0 (0-0)	0 (0-0)		
	CVB4	943-951	YQSHVLLAV	In silico	1.9 E-6	2 (0-5)	0 (0-100)		
	**CVB5**	**945-953**	**YQTHVLLAV**	**In silico**	**5.6 E-5**	**25 (9-47)**	**24 (7-55)**	**6.7 E-6**	**8 (0-55)**
	**CVB1-2-3-4-5-6**	**1246-1254**	**KLNSSVYSL**	**In silico**	**3.0 E-5**	**13 (1-36)**	**33 (0-70)**	**1.3 E-5**	**38 (0-83)**
	CVB1-2-3-4-5-6	1289-1297	QMVSSVDFV	In silico	2.7 E-6	1 (0-5)	40 (40-40)		
	CVB1-2-3-4-6	1307-1315	GILFTSPFV	In silico	1.0 E-7	0 (0-3)	0 (0-33)		
	CVB5	1311-1319	FLFTSPFVL	In silico	8.0 E-6	4 (1-14)	29 (20-56)		
	CVB1-2-3-4-5-6	1509-1517	KLFAGFQGA	In silico	1.0 E-7	0 (0-0)	0 (0-0)		
	CVB1-3	1585-1593	LMNDQEIGV	In silico	7.4 E-7	1 (0-12)	50 (0-100)		
	CVB2-4-5-6	1589-1598	ILMNDQEVGV	In silico	2.5 E-5	11 (1-892)	40 (24-99)	1.0 E-7	NA
	CVB1-5-6	1626-1634	FLAKEEVEV	In silico	1.0 E-7	0 (0-1)	0 (0-0)		
	CVB2-4	1631-1639	FLAREEAEV	In silico	5.3 E-6	2 (0-10)	34 (25-43)		
	**CVB1-2-3-4-5-6**	**1671-1679**	**RMLMYNFPT**	**In silico**	**2.8 E-5**	**24 (0-154)**	**25 (0-67)**	**6.2 E-6**	**16 (0-33)**
	CVB1-2-3-4-5-6	2107-2115	FLVHPVMPM	In silico	1.0 E-7	0 (0-0)	0 (0-0)		
	CVB1	804-813	RIYFKPKHVK	MS	4.3 E-5	16 (10-67)	66 (23-88)	1.0 E-7	NA
	**CVB1**	**1132-1140**	**KILPEVKEK**	**MS**	**2.0 E-5**	**9 (4-21)**	**58 (20-71)**		
	CVB1 only	*1132-1140*	*KILPEVKEK*	*MS*				*1.0 E-7*	*24 (20-29)*
	GAD only	*272-280*	*KMFPEVKEK*	*MS*				*1.0 E-7*	*23 (20-55)*
	**CVB1 x GAD**							**1.3 E-5**	**60 (23-100)**
	CVB1/CVB3	1233-1241/1236-1244	SVATNLIGR	MS	5.4 E-6	2 (1-6)	42 (0-83)		
HLA-A3 n=12 **n=4**	**CVB1/CVB3**	**1356-1364/1359-1367**	**KINMPMSVK**	**MS**	**1.0 E-5**	**4 (0-89)**	**95 (80-100)**	**3.1 E-5**	**98 (80-100)**
	CVB2	1361-1369	KVNMPMSVK	In silico	1.9 E-5	7 (0-15)	61 (40-100)		
	CVB3	1519-1528	GAYTGVPNQK	MS	1.0 E-7	0 (0-2)	0 (0-0)		
	**CVB3**	**1765-1774**	**VLRSGDPRLK**	**MS**	**1.4 E-5**	**6 (0-15)**	**68 (50-75)**	**1.8 E-5**	**78 (56-92)**
	**CVB5**	**760-768**	**SMFYDGWAK**	**In silico**	**1.9 E-5**	**12 (2-25)**	**33 (0-68)**	**2.9 E-5**	**33 (9-94)**
	CVB1-2-3-4-5-6	1204-1212	KMSNYIQFK	In silico	1.0 E-7	0 (0-0)	0 (0-0)		
	CVB1-2-3-4-5-6	2024-2032	IIIRTLMLK	In silico	4.4 E-6	2 (1-3)	0 (0-0)		
	CVB1-2-3-4-5-6	2027-2035	RTLMLKVYK	In silico	3.0 E-6	1 (0-6)	25 (16-33)		
	CVB2-3-4-5	2161-2169	KIRSVPVGR	In silico	1.0 E-7	0 (0-0)	0 (0-0)		

Table S2. Summary of T-cell screening and validation experiments (related to Fig. 2 and 3). The first columns detail the identity of each peptide. For the screening round (Fig. 2), $MMr^{+}CD8^{+}$ T-cell frequencies, MMr^{+} counts and percent effector/memory fractions are listed. For the more stringent validation round (Fig. 3), donors/peptides with <3 MMr^{+} cells counted were excluded from the final analysis, with frequencies noted as 1.0E-7 (i.e. 10^{-7}) and percent effector/memory fractions not assigned (NA). Merged table rows indicate peptides with high sequence homology. Validated epitopes are displayed in bold.

	nPOD case RRID SAMN#	Sex	Age (yrs)	T1D (yrs)	Positive aAbs	Pancreas VP1/qPCR/ Prot	HLA	Spleen							PLNs					PBMCs				
								CD8+	CVB MMr+		Flu MMr+		HCV/HIV MMr+		CD8+	CVB MMr+		Flu MMr+		CD8+	CVB MMr+		Flu MMr+	
								# $\times 10^3$	#	Freq	#	Freq	#	Freq	# $\times 10^{-3}$	#	Freq	#	Freq	# $\times 10^{-3}$	#	Freq	#	Freq
T1D (n=13)	6046-15879103	F	19	8	IA-2/ZnT8	+/+/+	A2<br>A3	173.0	2<br>8	1.2e-5<br>4.6e-5	na<br>33	na<br>1.9e-4	na<br>1	na<br>5.8e-6	na	na	na	na	na	na	na	na	na	na
	6052-15879109	M	12	1	IA/IA-2	+/+/−	A2	86.3	1	1.2e-5	0	0	0	0	na	na	na	na	na	na	na	na	na	na
	6195-15879251	M	19	5	IA/GAD/IA-2/ZnT8	−/+/+	A3	73.3	1	1.4e-5	12	1.6e-4	0	0	na	na	na	na	na	na	na	na	na	na
	6211-15879267	F	24	4	IA/GAD/IA-2/ZnT8	+/+/+	A2<br>A3	na	na	na	na	na	na	na	38.1	20*<br>1	5.4e-4*<br>2.7e-5	8	2.2e-4	46.7	14*<br>1	3.1e-4*<br>2.2e-5	14	3.1e-4
	6212-15879268	M	20	5	IA	+/+/−	A2	47.6	0	0	8	1.7e-4	1	2.1e-5	na	na	na	na	na	na	na	na	na	na
	6224-15879280	F	21	2	None	−/na/na	A2	30.2	0	0	3	1.0e-4	0	0	na	na	na	na	na	na	na	na	na	na
	6243-15879299	M	13	5	IA	+/na/na	A2	122.8	0	0	43	3.5e-4	2	1.6e-5	na	na	na	na	na	na	na	na	na	na
	6265-15879319	M	11	8	IA/GAD	−/−/na	A3	126.2	13	1.0e-4	1	7.9e-6	1	7.9e-6	na	na	na	na	na	na	na	na	na	na
	6325-15879379	F	20	6	IA/GAD/IA-2	+/−/+	A2	128.4	1	7.8e-6	2	1.6e-5	0	0	na	na	na	na	na	na	na	na	na	na
	6362-15879415	M	25	0	GAD	+/+/+	A3	40.5	6	1.5e-4	17	4.2e-4	0	0	125.7	29<br>25*	2.3e-4<br>2.0e-4*	54	4.3e-4	na	na	na	na	na
	6438-15879491	F	39	10	GAD	na	A2	220.2	3	1.4e-5	166	7.5e-4	0	0	na	na	na	na	na	na	na	na	na	na
	6456-15879509	F	30	0	GAD/ZnT8	−/na/na	A2	118.2	0	0	22	1.9e-4	0	0	na	na	na	na	na	na	na	na	na	na
	6480-15879533	M	17	3	IA/IA-2	na	A3	17.5	3	1.7e-4	0	0	3	1.7e-4	na	na	na	na	na	na	na	na	na	na
aAb+ (n=10)	6080-15879137	F	69	na	IAA/GAD	+/+/−	A2	9.6	0	0	1	1.0e-4	2	2.1e-4	na	na	na	na	na	na	na	na	na	na
	6151-15879207	M	30	na	GAD	−/−/−	A2	97.4	1	1.0e-5	10	1.0e-4	0	0	na	na	na	na	na	na	na	na	na	na
	6156-15879212	M	40	na	GAD	−/+/na	A3	45.3	0	0	13	2.9e-4	1	2.2e-5	na	na	na	na	na	na	na	na	na	na
	6158-15879214	M	40	na	IAA/GAD	+/+/−	A3	15.1	0	0	0	0	0	0	na	na	na	na	na	na	na	na	na	na
	6171-15879227	F	4	na	GAD	−/−/na	A2	35.1	4	1.1e-4	0	0	0	0	87.3	1<br>11*	1.2e-5<br>1.3e-4*	32	3.7e-4	19.3	9<br>5*	5.0e-4<br>2.8e-4*	42	2.2e-3
	6181-15879237	M	32	na	GAD	−/+/na	A3	70.0	0	0	61	8.7e-4	1	1.4e-5	na	na	na	na	na	na	na	na	na	na
	6197-15879253	M	22	na	GAD/IA-2	+/+/−	A2	87.1	8	9.2e-5	2	2.3e-5	1	1.1e-5	38.1	0<br>25*	0<br>6.8e-4	7	1.9e-4	na	na	na	na	na
	6347-15879401	M	9	na	IAA	na	A2	97.4	1	1.0e-5	5	5.1e-5	1	1.0e-5	na	na	na	na	na	na	na	na	na	na
	6421-15879474	M	7	na	GAD	+/−/na	A2<br>A3	129.8	2<br>2	1.5e-5<br>1.5e-5	na<br>2	na<br>1.5e-5	na<br>2	na<br>1.5e-5	na	na	na	na	na	na	na	na	na	na
	6429-15879482	M	22	na	IAA/GAD	−/−/+	A2	41.4	0	0	1	2.4e-5	0	0	na	na	na	na	na	na	na	na	na	na
ND (n=15)	6102-15879159	F	45	na	None	+/−/−	A3	19.6	0	0	20	1.0e-3	0	0	na	na	na	na	na	na	na	na	na	na
	6140-15879197	M	38	na	None	+/−/−	A3	17.0	0	0	1	5.9e-5	4	2.4e-4	na	na	na	na	na	na	na	na	na	na
	6338-15879392	M	17	na	None	na	A2<br>A3	134.8	9*<br>0	6.7e-5*<br>0	5	3.8e-5	na	na	173.3	3*<br>2	1.7e-5*<br>1.2e-5	3	1.7e-5	na	na	na	na	na
	6368-15879421	M	38	na	None	+/na/na	A3	307.7	10	3.2e-5	972	3.2e-3	2	6.5e-6	na	na	na	na	na	na	na	na	na	na
	6386-15879439	M	14	na	None	na/na/−	A2<br>A3	330.2	48*<br>4	1.5e-4<br>1.2e-5	59	1.8e-4	na	na	674.0	54*<br>9	8.1e-5*<br>1.4e-5	74	1.1e-4	na	na	na	na	na
	6420-15879473	M	11	na	None	na	A2<br>A3	394.3	18*<br>1	4.6e-5*<br>2.6e-6	19	4.9e-5	na	na	na	na	na	na	na	120.0	9*<br>0	7.6e-5*<br>0	7	5.9e-5
	6461-15879514	M	14	na	None	na	A2<br>A3	75.0	44*<br>3	6.0e-4*<br>4.1e-5	9	1.2e-4	na	na	37.3	33*<br>2	9.1e-4*<br>5.5e-5	11	3.0e-4	na	na	na	na	na
	6488-15879541	F	5	na	None	na	A2<br>A3	540.0	1<br>23	1.9e-6<br>4.4e-5	4	7.6e-6	na	na	na	na	na	na	na	na	na	na	na	na

**HDL-2**	F	7	na	None	na	A2	189.1	**6**<br>**3**	**3.3e-5***<br>**1.7e-5**	**3**	**1.7e-5**	na	na	na	na	na	na	na	na	na	na	na	na
HDL-4	M	2	na	None	na	A2	77.2	1<br>0	**1.3e-5***<br>0	1	**1.3e-5**	na	na	na	na	na	na	na	na	na	na	na	na
**HDL-5**	M	5	na	None	na	A2	108.1	**5**<br>**11**	**4.8e-5***<br>**1.1e-4**	2	**1.9e-5**	na	na	na	na	na	na	na	na	na	na	na	na
**HDL-19**	M	7	na	None	na	A2	106.7	**7**<br>1	**6.9e-5***<br>9.9e-6	2	**2.0e-5**	na	na	na	na	na	na	na	na	na	na	na	na
**HDL-24**	M	2	na	None	na	A2	40.8	**7**<br>0	**1.8e-4***<br>0	1	**2.6e-5**	na	na	na	na	na	na	na	na	na	na	na	na
**HDL-25**	F	2	na	None	na	A2<br>A3	59.8	1<br>**4**	**17e-5***<br>**6.9e-5**	**11**	**1.9e-4**	na	na	na	na	na	na	na	na	na	na	na	na
**HDL-27**	F	0.2	na	None	na	A2	64.1	**10**<br>0	**1.7e-4***<br>0	0	0	na	na	na	na	na	na	na	na	na	na	na	na

Table S3. Spleen, PLN and PBMC specimens analyzed by flow cytometry MMr staining (related to Fig. 4). Results available in the nPOD repository are reported for pancreas VP1 staining, Enterovirus qPCR and CVB proteomics (prot) analyses. The last 3 sets of columns detail the number of total CD8$^+$ T cells analyzed and the MMr$^+$ counts and frequencies for CVB peptides, Flu peptides (Flu MP$_{58\text{-}66}$ for HLA-A2, Flu NP$_{265\text{-}273}$ for HLA-A3) and naïve viral peptides (HCV PP$_{1406\text{-}1415}$ for HLA-A2, HIV nef$_{84\text{-}92}$ for HLA-A3). HLA-A2-restricted CVB1-2-3-4-5-6$_{1246\text{-}1254}$ and CVB1$_{271\text{-}279}$ and HLA-A3-restricted CVB1$_{1356\text{-}1364}$/CVB3$_{1359\text{-}1367}$ and CVB1$_{1132\text{-}1140}$ were analyzed. For CVB1$_{271\text{-}279}$ and CVB1$_{1132\text{-}1140}$, the corresponding values are marked with an asterisk. Positive results (≥3 MMr$^+$ counts and $\geq 10^{-5}$ frequency) are shown in bold (and underlined for CVB). na, not applicable or not available. The non-diabetic donor group comprised specimens from nPOD and HANDEL repositories (HDL codes). Results obtained with the two sets of specimens were similar and were therefore pooled for figure representation.

	nPOD case RRID SAMN#	Sex	Age (yrs)	T1D (yrs)	Positive aAbs	Pancreas VP1/qPCR/ Prot	HLA	Spleen						PLNs					
								CVB MMr+		CMV/Flu MMr+		WNV MMr+		CVB MMr+		CMV/Flu MMr+		WNV MMr+	
								#	Density	#	Density	#	Density	#	Density	#	Density	#	Density
T1D (n=8)	6046-15879103	F	19	8	IA-2/ZnT8	+/+/+	A2<br>A3	64	1.40	35	0.70	18	0.94	45	8.97	29	6.08	19	4.21
	6052-15879109	M	12	1	IA/IA-2	+/+/−	A2	134	3.18	93	1.46	11	0.19	182	14.46	130	10.88	25	1.99
	6211-15879267	F	24	4	IA/GAD/IA-2/ZnT8	+/+/+	A2<br>A3	85	1.37	78	1.36	25	0.88	133	3.22	215	6.17	58	1.43
	6224-15879280	F	21	2	None	−/na/na	A2	165	1.77	43	0.40	4	0.03	35	4.16	43	5.07	5	0.56
	6265-15879319	M	11	8	IA/GAD	−/−/na	A3	128	0.83	78	0.64	13	0.42	17	2.92	48	8.75	11	1.95
	6325-15879379	F	20	6	IA/GAD/IA-2	+/-/+	A2	83	1.92	35	0.55	19	0.32	188	7.80	57	4.64	18	1.40
	6438-15879491	F	39	10	GAD	na	A2	153	3.70	99	1.72	4	0.07	84	12.80	82	5.57	25	0.54
	6480-15879533	M	17	3	IA/IA-2	na	A3	9	0.44	13	0.30	7	0.37	49	2.95	46	2.91	10	0.76
AAb+ (n=2)	6158-15879214	M	40	na	IA/GAD	+/+/−	A3	159	13.81	40	0.79	14	0.23	67	8.13	70	4.18	20	1.12
	6197-15879253	M	22	na	GAD/IA-2	+/+/−	A2	48	5.89	54	4.30	11	0.48	157	20.30	145	22.91	56	7.92
ND (n=5)	6102-15879159	F	45	na	None	+/−/−	A3	na	na	18	0.38	10	0.34	55	3.73	32	2.25	18	1.08
	6227-15879283	F	17	na	None	+/na/+	A2<br>A3	53<br>36	1.20<br>0.50	45<br>74	0.89<br>0.64	4<br>12	0.04<br>0.35	99<br>16	19.18<br>3.02	55<br>132	10.13<br>23.06	13<br>37	7.24<br>2.55
	6271-15879325	M	17	na	None	−/na/−	A2	82	4.36	53	2.04	10	0.20	3	5.97	45	8.55	5	1.05
	6232-15879288	F	14	na	None	+/na/na	A2	66	1.75	16	0.26	6	0.09	66	4.61	24	1.93	6	0.55
	6368-15879421	M	38	na	None	+/na/na	A3	20	0.53	22	0.26	9	0.45	166	10.79	110	7.93	71	5.20

Table S4. nPOD spleen and PLN specimens analyzed by *in-situ* MMr staining (related to Fig. 5). Results available in the nPOD repository are reported for pancreas VP1 staining, Enterovirus qPCR and CVB proteomics (prot) analyses. The last 2 sets of columns detail the number of MMr^+ cells counted and densities per mm^2 for CVB peptides, CMV/Flu peptides and naïve West Nile Virus (WNV) peptides (detailed in Fig. S7). na, not applicable or not available. For HLA-A2/A3$^+$ cases 6211 and 6046, HLA-A3-restricted peptides were studied.

Movie S1. Kinetics of CVB infection and CVB-eGFP transfer through filopodia in ECN90 β cells. Real-time imaging of ECN90 β cells infected with CVB-eGFP at MOI 100 and stained with Cytotox Red to visualize dead cells. The movie shows an intact β cell in contact with the filopodia of an infected eGFP$^+$ cell, turning eGFP$^+$ at and subsequently protruding filopodia before dying (Cytotox Red$^+$). Infected cells are labeled in green, dead cells are labeled in red.

Movie S2. Death kinetics and morphology in CVB-infected ECN90 β cells and definition of a single-cell analysis mask. β cells (infected at 300 MOI, in the absence of T cells) are labeled in red, dead cells are labeled in green, merged images of dead β cells are labeled in yellow. Contours in magenta red indicate the areas of single-cell death defined by the imaging software based on the preset analysis mask.

Movie S3. Death kinetics and morphology of ECN90 β cells co-cultured with CVB-reactive CD8$^+$ T-cell transductants and definition of a cell cluster analysis mask. β cells (pulsed with 1 µM CVB1$_{1356\text{-}1364}$ peptide, without infection) are labeled in red, dead cells are labeled in green, merged images of dead β cells are labeled in yellow. Contours in magenta red indicate the areas of clustered cell death defined by the imaging software based on the preset analysis mask.

Movie S4. Mask analyses for counting of single-cell death areas of CVB-infected ECN90 β cells co-cultured with CVB-reactive CD8$^+$ T-cell transductants. β cells (infected at 300 MOI and co-cultured at a 1:1 T:β-cell ratio) are labeled in red, dead cells are labeled in green, merged images of dead β cells are labeled in yellow. The analysis with the defined single-cell analysis mask is shown, with contours in magenta red indicating the areas counted with each mask. See also Movie S5 for the analysis of the same field with the defined cluster cell analysis mask.

Movie S5. Mask analyses for counting of cluster cell death areas of CVB-infected ECN90 β cells co-cultured with CVB-reactive CD8$^+$ T-cell transductants. β cells (infected at 300 MOI and co-cultured at a 1:1 T:β-cell ratio) are labeled in red, dead cells are labeled in green, merged images of dead β cells are labeled in yellow. The analysis with the defined cluster cell analysis mask is shown, with contours in magenta red indicating the areas counted with each mask. See also Movie S4 for the analysis of the same field with the defined single-cell analysis mask.
